# Protecting nature in diverse ways: The socio-demographic spread of benefits from connecting with nature

**DOI:** 10.1007/s13280-025-02233-6

**Published:** 2025-08-21

**Authors:** Kate Sollis, Lily van Eeden, Usitha Rajeevan, Kate Lee, Brenda Lin, Lucy Keniger, Pauline Marsh, Emily Flies

**Affiliations:** 1 Geography, Planning and Spatial Sciences, University of Tasmania, Sandy Bay Campus, Hobart, TAS 7001 Australia; 2 RMIT University STEM College, Applied Chemistry and Environmental Sciences Melbourne, Melbourne, VIC 3000 Australia; 3 Department of Energy, Environment, and Climate Action, Government of Victoria, Arthur Rylah Institute for Environmental Research, Heidelberg, VIC Australia; 4 School of Agriculture, Food and Ecosystem Sciences, University of Melbourne, Parkville, VIC Australia; 5 BehaviourWorks Australia, Monash University, Clayton, VIC Australia; 6 CSIRO, Land & Water Ecosystem Sciences Precinct, 41 Boggo Road, Dutton Park, QLD 4102 Australia; 7 Wicking Dementia Research and Education Centre, University of Tasmania, Sandy Bay Campus, Hobart, TAS 7001 Australia

**Keywords:** Environmental stewardship, Gardening, Life satisfaction, Nature connection, Pro-environmental behaviours

## Abstract

Nature connection and its relationships with stewardship vary across social and environmental contexts. Through a nationally representative survey in Australia, this study examined engagement in four different pro-environmental behaviours (PEBs) (advocacy, consumer-conscious, conservation, and gardening), their socio-demographic correlates, and the associations with both nature connection and well-being. We found strong associations between nature connection and all examined PEBs; however, different groups were more likely to engage in different types of PEBs. For example, younger adults are more likely to engage in advocacy and conservation behaviours, while older Australians are more likely to engage in consumer-conscious behaviours and gardening. All PEBs were significantly associated with well-being; however, only the advocacy behaviours variable remained significant when including nature connection as a control variable. These findings highlight the importance of biodiversity policies that holistically enhance nature connection for a range of people, alongside sustainability measures to support the well-being of both people and planet.

**Supplementary Information:**

The online version contains supplementary material available at 10.1007/s13280-025-02233-6

## Introduction

Humanity is currently on an unsustainable trajectory, having already transgressed six of the nine planetary boundaries (Richardson et al. 2023) and experiencing social, climate and biodiversity crises (Pörtner et al. 2023). These conditions are the result of complex social, cultural, economic and ecological systems which are difficult to change. However, learnings from system science suggest that several leverage points exist, where small changes can have big impacts. Two such ‘deep’ leverage points for change are mindsets and paradigms (Meadows 1999). The relationship between humans and the rest of non-human nature (sometimes called human–nature connection, HNC) is related to both mindset and paradigm. Moreover, nature connection has positive links with both our individual well-being (Capaldi et al. 2014; Sollis et al. 2024) and our propensity to undertake pro-environmental behaviours (PEBs) (Mackay and Schmitt 2019; Martin et al. 2020). For these reasons combined, human–nature connection has been identified as a crucial leverage point for sustainability (Ives et al. 2018; Riechers et al. 2021).

Like most relationships, HNC is complex and multidimensional, which can make it difficult to quantify. HNC is broadly defined as the ‘extent to which humans see themselves as part of nature’ (Barragan-Jason et al. 2022, p. 2) and is often described using other terms, including ‘nature relatedness’ (Nisbet et al. 2009) and ‘connectedness to nature’ (Mayer and Frantz 2004). Despite HNC being a difficult-to-measure construct, numerous measurement tools have been developed, largely in the field of environmental psychology, to assess the HNC among population groups. These measures are often multidimensional and include cognitive, affective, emotive, philosophical, material, experiential, and behavioural elements (Ives et al. 2017; Hatty et al. 2020; Barragan-Jason et al. 2022; Sollis et al. 2025) to capture an individual’s sense of their relationship with the natural world (Richardson and Thatcher 2024).

One multidisciplinary review identified HNC research as falling into three broad clusters: HNC as place, HNC as mind and HNC as experience (Ives et al. 2017). Others have tried to categorise HNC by five dimensions: immediateness, consciousness, intentionality, degree of human mediation and direction of outcomes for humans and non-humans. Research in this field is further complicated by the diverse definitions and conceptualisations of ‘nature’, which are influenced by culture and history (Ducarme and Couvet 2020) and here we take to mean ‘the vast array of all non-human living and non-living things’.

There has been a significant increase in research interest in HNC across disciplines over the last decade (Ives et al. 2017; Richardson and Thatcher 2024). As such, numerous validated scales have been developed to measure human–nature connection including Inclusion of Nature in Self (INS) (Schultz 2001), Connectedness to Nature Scale (Mayer and Frantz 2004), the Nature Relatedness Scale (Nisbet et al. 2009) and the 12-question Connection with Nature scale (CN-12) (Hatty et al. 2020). These scales vary in how they conceptualise and measure nature connection (Sollis et al. 2025). For example, the INS is a unidimensional, single item, pictorial tool used to measure the extent to which an individual includes nature as part of their identity (Schultz 2001; Kleespies et al. 2021). The INS is a self-reporting tool that assesses nature connection as a cognitive construct of the individual. Conversely, The CN-12 is a multidimensional tool comprised of 12 Likert-scale questions spanning three dimensions (identity, experience and philosophy) (Hatty et al. 2020). Despite variation across the breadth of HNC measurement tools available, studies have found that they are highly correlated to each other (Tam 2013; Hatty et al. 2020) although multidimensional tools may be more appropriate for measuring different aspects of HNC (Tam 2013).

Many studies have examined the link between a person’s level of nature connection and the actions they take to support environments (i.e. pro-environmental behaviours, PEBs) including conservation and restoration behaviours that directly result in positive outcomes for species, biodiversity and ecosystems (Barbett et al. 2020; van Eeden et al. 2024). PEBs have been variously categorised into different subgroups that generally align with civic engagement, such as activism or advocacy (e.g. involvement with demonstrations and other campaigning) and non-activist public sphere behaviours (e.g. citizenship actions like donating to pro-environmental organisations, voting for pro-environmental governments), and private sphere or personal practice behaviours (e.g. sustainable consumption, wildlife-friendly gardening) (Stern 2000; Alisat and Riemer 2015). Private sphere behaviours generally have more direct benefit to the environment than civic engagement behaviours, but the latter may be more focused on system-level change (Stern 2000). These categorisations are useful for understanding and influencing behaviours because they may be associated with different demographic and socio-psychological predictors (Alisat and Riemer 2015; Pisano and Lubell 2015). For example, personal practices are often related to individual characteristics including demographics, beliefs and attitudes, while activist behaviours are more likely to be linked to contextual factors such as social networks and local environmental problems (Alisat and Riemer 2015). Such relationships have also been identified for private sphere behaviours (Iwińska et al. 2023).

Positive associations between various types of PEBs and human–nature connection have been identified in the literature (e.g. Whitburn et al. 2020; Sockhill et al. 2022). Nature conservation behaviours, which include pro-biodiversity behaviours, have shown positive associations with nature engagement (Soga and Gaston 2023). Additionally, simple activities in nature such as smelling wildflowers, collecting shells and listening to bird songs have found to be associated with nature connection (Richardson et al. 2020). This link is bi-directional, with a stronger connection to nature also likely to result in stronger action for the environment, including environmental activism (Lewis and Townsend 2015).

For example, an Australian study found that an increase in nature connection was associated with greater engagement in both public and private sphere PEBs, such as cleaning up litter in a public place, donating money to organisations working for the environment, and choosing to plant native species for planting or gardening (Meis-Harris et al. 2019). Furthermore, there is evidence of nature connection positively influencing environmentally friendly travel choices (Mandić et al. 2023). It is also been shown that despite having different values (ecocentric vs anthropocentric), people with higher nature connection engage more in pro-biodiversity behaviours, energy and water conservation behaviours and are more supportive of biodiversity-focussed policies compared to those who have lower connection to nature (Sockhill et al. 2022). This positive association is also evident for children’s pro-ecological behaviours, such as recycling and waste separation (Barrera-Hernández et al. 2020).

In addition, clear links have also been made in the literature between nature connection and well-being, defined broadly as measures assessing life satisfaction, eudaimonic well-being, and mental health (Dean et al. 2018; Richardson and Hamlin 2021; Barragan-Jason et al. 2023). However, few studies have looked at the relationship between well-being and PEBs. Of those that have one found a positive association between well-being and PEB in seven different countries regardless of the country’s economic status (Capstick et al. 2022). This study, however, did not consider the role of nature connection in the relationship between well-being and PEBs. Other studies that investigated the relationship between PEBs and well-being reported both positive (Mandić et al. 2023) and negative (Ibáñez-Rueda et al. 2020; Whelan et al. 2022) relationships.

This study examines PEBs (defined through four different measures: advocacy and other public sphere behaviours; consumer-conscious behaviours; conservation behaviours; and gardening and lawncare) and their links to nature connection and well-being (measured through life satisfaction), in a nationally representative survey in Australia. We contribute to the existing literature base in three ways. Firstly, we examine the overall level of engagement in PEBs in Australia, as well as exploring the correlates of engaging in these behaviours. Secondly, we explore the extent to which engagement in PEBs is related to nature connection, and how this varies across demographics in Australia. Finally, we examine the relationship between engagement in PEBs and well-being. To achieve a nature connected Australia is the #1 goal in Australia’s Strategy for Nature (Commonwealth of Australia 2019). This study makes an invaluable contribution to the evidence base both in Australia and internationally as to how enhancing nature connection can result in benefits for both the environment and human well-being.

## Materials and methods

### Survey design

The survey was enumerated using an existing panel sample run by the Online Research Unit, an Australian online data collection agency, between 6 and 31 July 2023. The survey comprised of 15 questions related to the individual’s demographics, five questions on human–nature connection, four questions on nature engagement and environmental behaviours, two questions on well-being (the Personal Well-being Index, a life satisfaction measure, and the AQoL-6D, a HRQoL measure), and six questions on nature connection and well-being across different environments (see Supplementary Information for full survey). This project was approved by the University of Tasmania Human Research Ethics Committee (project ID 28109). All participants provided informed consent to being involved in the survey.

Nature connection was assessed using the CN-12, which consists of 12 questions evaluating nature connection across three dimensions: identity, experience, and philosophy (Hatty et al. 2020). Scores for the items were averaged using a mean to derive an overall score, as were the scores for items specific to each dimension. As a robustness check, two additional measures of nature connection and engagement—the INS and nature contact—were also included in the analysis. The CN-12 was selected as the primary variable for assessing nature connectedness due to its development and validation within the Australian context.

The Personal Well-being Index (PWI), a commonly used measure of life satisfaction, was used to examine the relationship between engagement in PEBs and well-being. The PWI measures life satisfaction in seven domains of life: standard of living, health, achieving in life, personal relationships, safety, sense of community, and future security (with religion being an optional domain) (International Wellbeing Group, 2024).

A broad range of PEBs were represented through four separate measures:

1. Advocacy and other public sphere behaviours: Reflected through a scale of nine items. This scale is adapted based on the Victorians Value Nature survey (Squires et al. 2022) and was used to allow comparisons within the Australian context. The scale asked how often individuals engage in a range of different types of advocacy and public sphere behaviours, with responses options on a 5-point Likert scale ranging from ‘never’ to ‘very often’. A mean was calculated for these nine items to obtain an aggregate score.
2. Consumer-conscious behaviours: A single-item scale asking how often individuals engage in consumer-conscious behaviours on a 5-point Likert scale ranging from ‘never’ to ‘very often’.
3. Conservation activities: Respondents were asked about different activities they have engaged in over the last year or two in a range of different environments.^1^ One of these activities was ‘engage in activities to protect or care for nature’.^2^ Examples were provided in the survey to prompt respondents, such as landcare, conservation, coast care, marine debris cleanup, starfish removal, and create wildlife habitat.
4. Gardening and lawncare: Another activity asked about for the different environments was ‘gardening or lawncare activities’.^3^

### Sample

An initial sample of 4114 individuals participated in the survey, with 108 responses excluded following a data cleaning process including cases where participants had completed the survey too quickly (less than 20% of the median completion time) and those who consistently gave identical responses across multiple questions (‘straight-liners’). The final sample consisted of 4006 de-identified individuals from 79 998 individuals who were invited to participate. This reflects a response rate of 5% which is typical for large online panels (Daikeler et al. 2020).

The sampling strategy aimed to achieve demographic diversity by setting quotas to ensure adequate representation across Australian states and territories (approximately 500 participants per region) and between urban and rural areas (50% from capital cities, 25% from major cities outside capitals, and 25% from other regions). This involved promoting the survey to target demographics until the quota was reached. It should be noted that the quota for the Northern Territory was not fully met with only 306 responses received, aligning with historical challenges in survey participation in that region (Australian Bureau of Statistics 2022). Additionally, quotas for educational levels were established to ensure representation across different groups. Overall, the sample demonstrated balanced representation across key demographics such as gender, age, education, and income (see Table S1, Supplementary Information).

### Analysis approach

Descriptive statistics were firstly generated to better understand the extent to which respondents engaged in different types of PEBs. For the advocacy and other public sphere behaviours scale and consumer-conscious behaviours measure, the percentage of individuals reporting that they engaged in each behaviour at least ‘sometimes’ was calculated. For the measures on conservation activities and gardening/lawncare, the percentage of respondents who reported that they had engaged in this activity in any environment in the last year or two was tabulated. Descriptive statistics were also produced for the different environments in which people engaged in conversation activities and gardening/lawncare.

Ordinary Least Squares (OLS) and logit models were produced to examine the socio-demographic correlates of PEB engagement, and to examine the associations between nature connection and PEBs. The modelling focussed on three separate areas:

1. Socio-demographic correlates of PEB engagement (with the four PEBs listed above as outcome variables, and all demographics being predictor variables)
2. The association between nature connection and PEB engagement (the four PEBs listed above as outcome variables, the CN-12 as the predictor variable and socio-demographic variables as controls)
3. The association between PEB engagement and life satisfaction (the PWI as the outcome variable, the four PEBs listed above as predictor variables, and socio-demographic variables as controls)

Where advocacy and other public sphere behaviours, and the PWI was the outcome variable, OLS regression was used. An ordered logit model was used for the model with consumer-conscious behaviours as the outcome variable, while logit models were produced for modelling for the other two outcome variables (conservation behaviours, and gardening and lawncare). In our analysis, a 5% significance level is used (associations which are significant at the 10% level are noted in tables but not considered significant). StataSE 17 was used for analysis.

Socio-demographic variables collected in the survey were used as control variables, including: age, gender, whether an individual identified as Aboriginal and/or Torres Strait Islander, whether the individual self-identified as having a disability, language spoken at home, employment status, highest level of education, personal income, socioeconomic status of area, state of current location, remoteness of current location, and remoteness level respondent grew up in. To test for multicollinearity among the independent variables, variance inflation factors (VIFs) were calculated. These ranged from 1.02 to 4.58 (mean value 1.79, indicating low multicollinearity (Table S2, Supplementary Information).

## Results

### Engagement in PEBs

In Australia, individuals have engaged in PEBs to varying degrees (Table 1). The most frequent was consumer-conscious behaviours, with 78% of participants reporting that they consider environmental impacts when making purchases at least sometimes. Other types of advocacy behaviours were frequently engaged with, with many participants bringing up positive nature experiences in conversation (69%), voting for people or parties that support nature (68%), and encouraging others to change an environmentally harmful behaviour (65%). Engaging in conservation activities was one of the least common activities (18%). A total of 69% of individuals reported having engaged in gardening (or ‘lawncare’) activities in the past two years.

**Table 1:** Engagement in different PEBs

PEBs	% undertaking this activity at least sometimes
*Advocacy and other public sphere behaviours*	
I bring up positive nature experiences in a conversation, such as interesting facts about wildlife or stories about things I’ve seen or done in nature	69.2%
I vote for people, parties, or policies that support nature	67.8%
I try to encourage others to change a behaviour that I think is harmful to the environment	65.1%
I bring up environmental issues in conversation with my peers	58.3%
I sign petitions about environmental issues I’m concerned about	47.5%
I donate money to organisations that protect or support the environment	38.7%
I share articles, pictures, or videos on social media about nature or environmental issues	37.6%
I contact businesses or governments about impacts on environmental issues I’m concerned about	19.8%
I attend protests or rallies related to environmental issues	14.5%
*Consumer-conscious behaviours*	
I consider environmental impacts when making purchasing decisions (e.g. product/service waste, packaging, carbon footprint, sustainability, local source etc.)	78.1%
*Conservation behaviours*	
Engage in activities to protect or care for nature*	18.12%
*Gardening and lawncare*	
Engage in gardening or lawncare activities*	69.18%

*This question was part of a separate scale, which asked whether individuals had engaged in these activities at least once in the last two years within a range of different environments

**Fig. 1:**
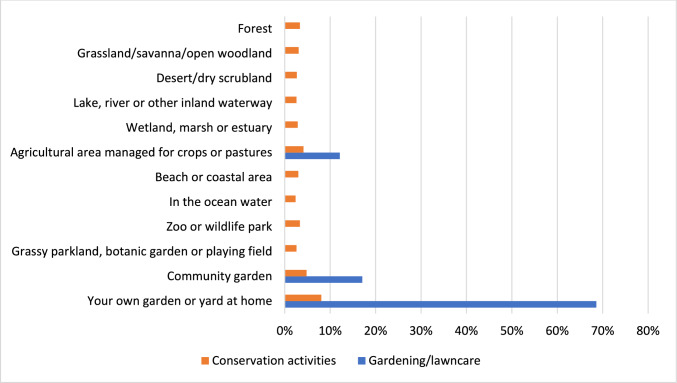
Percentage of respondents engaging in conservation activities and gardening/lawncare by environment. Note: Gardening/lawncare was an option for only three environments: agricultural area, community garden, and your own garden or yard at home

For conservation behaviours and gardening/lawncare we also asked where these behaviours took place (Fig. 1). ‘Your own garden or yard at home’ was the most common environment for both conservation activities and gardening/lawncare, followed by community garden and agricultural area.

### Engagement in PEBs for different population groups

Analysis of the socio-demographic correlates of the four outcome measures indicates that certain groups were more likely to engage in PEBs (Table 2). Those who identify as female were more likely to engage in advocacy (*p* < 0.01), consumer-conscious (*p* < 0.01), and conservation behaviours (*p* < 0.05) regardless of their other circumstances (age, education level, income, employment, childhood environment, etc.). Indigenous Australians were more likely to engage in advocacy (*p* < 0.01). Those with disability/ies were more likely to engage in advocacy (*p* < 0.01) and consumer-conscious behaviours (*p* < 0.05). People who speak a language other than English were more likely to engage in advocacy (*p* < 0.01). People aged 18–50 were more likely to engage in conservation behaviours (*p* < 0.01) but less likely to engage in gardening/lawncare (*p* < 0.01) compared to those aged over 70. Those aged over 70 were more likely to engage in consumer-conscious behaviours than those aged 18–50 (*p* < 0.01) and those aged 51–70 (*p* < 0.05). Those who grew up in a rural/regional area were more likely to engage advocacy (*p* < 0.01), consumer-conscious (*p* < 0.05), and conservation behaviours (*p* < 0.05). Other socio-demographic characteristics important in predicting engagement in some PEBs included education level, employment status, income quintile, socio-economic status of area, state/territory, and remoteness of current location (see Table 2).

**Table 2:** Determinants of engaging in PEBs

VARIABLES	(1)	(2)	(3)	(4)
	Advocacy behaviours	Consumer-conscious behaviours	Conservation behaviours	Gardening and lawncare
*Age*				
18–30 years	0.0223	− 0.406***	0.943***	− 0.922***
	(0.0659)	(0.149)	(0.233)	(0.266)
31–50 years	− 0.0202	− 0.499***	0.663***	− 0.433**
	(0.0625)	(0.141)	(0.226)	(0.204)
51–70 years	− 0.0700	− 0.272**	0.324	− 0.0585
	(0.0530)	(0.119)	(0.200)	(0.168)
*Gender*				
Identifies as female	0.122***	0.477***	0.202**	0.0616
	(0.0288)	(0.0657)	(0.0935)	(0.107)
Non-binary	0.478*	1.127*	1.128*	
	(0.258)	(0.578)	(0.669)	
Identifies as Aboriginal and/or Torres Strait Islander	0.336***	0.394*	0.413	0.809
	(0.0987)	(0.223)	(0.275)	(0.575)
Has disability/ies	0.176***	0.288**	0.242	− 0.126
	(0.0514)	(0.117)	(0.162)	(0.173)
Speaks language other than English at home	0.171***	0.117	0.0635	− 0.0483
	(0.0488)	(0.109)	(0.150)	(0.184)
*Highest level of education*				
Has not completed high school (Year 12)	− 0.116*	− 0.292**	− 0.477**	− 0.412*
	(0.0627)	(0.143)	(0.231)	(0.216)
Certificate/Diploma	0.0752*	0.196*	0.0538	0.0574
	(0.0456)	(0.103)	(0.146)	(0.174)
Undergraduate	0.239***	0.332***	0.155	0.113
	(0.0467)	(0.105)	(0.147)	(0.181)
Postgraduate	0.327***	0.592***	0.131	− 0.00459
	(0.0498)	(0.113)	(0.160)	(0.187)
*Employment status*				
Part-time	0.0531	0.0684	− 0.116	0.218
	(0.0473)	(0.107)	(0.149)	(0.179)
Casual	0.0121	− 0.0169	0.0517	0.150
	(0.0634)	(0.142)	(0.192)	(0.263)
Self-employed	− 0.0132	0.157	0.0286	0.102
	(0.0603)	(0.139)	(0.196)	(0.213)
Engaged in home duties/volunteer work	− 0.0521	− 0.0772	− 0.0140	0.804***
	(0.0686)	(0.155)	(0.213)	(0.274)
Retired	− 0.0595	− 0.0611	− 0.0791	0.405**
	(0.0533)	(0.120)	(0.184)	(0.179)
Not working/studying	− 0.0492	− 0.0780	− 0.252	− 0.144
	(0.0875)	(0.200)	(0.291)	(0.305)
Student only	0.268**	0.508**	0.618**	0.217
	(0.115)	(0.252)	(0.303)	(0.580)
*Personal income quintile*				
Lowest income quintile	0.00392	− 0.0900	− 0.203	− 0.190
	(0.0535)	(0.121)	(0.172)	(0.195)
2nd-lowest income quintile	0.0171	0.0346	− 0.159	− 0.194
	(0.0468)	(0.105)	(0.147)	(0.174)
4th-highest income quintile	− 0.0519	0.122	− 0.161	0.109
	(0.0443)	(0.0989)	(0.136)	(0.170)
Highest income quintile	− 0.187***	− 0.0236	− 0.486***	− 0.0292
	(0.0505)	(0.113)	(0.161)	(0.190)
Standardised IRSAD score	0.0375**	0.0527	− 0.00709	− 0.158**
	(0.0182)	(0.0411)	(0.0587)	(0.0653)
*State/territory*				
Victoria	0.0113	0.0394	0.0512	0.231
	(0.0538)	(0.123)	(0.172)	(0.178)
Queensland	− 0.0423	− 0.111	− 0.212	0.0453
	(0.0538)	(0.123)	(0.181)	(0.176)
South Australia	− 0.000658	0.313**	− 0.0257	0.444**
	(0.0545)	(0.124)	(0.177)	(0.194)
Western Australia	0.00273	0.225*	0.135	0.0783
	(0.0542)	(0.123)	(0.173)	(0.182)
Tasmania	− 0.0161	0.156	− 0.107	0.116
	(0.0580)	(0.132)	(0.187)	(0.228)
Australian Capital Territory	0.00810	0.356***	0.0614	0.464**
	(0.0581)	(0.132)	(0.187)	(0.209)
Northern Territory	0.143**	0.153	0.00500	0.171
	(0.0684)	(0.155)	(0.217)	(0.287)
*Current remoteness level*				
Regional	0.0199	0.194**	0.0187	0.527***
	(0.0412)	(0.0932)	(0.134)	(0.151)
Remote	− 0.0731	0.0352	0.230	0.417
	(0.0835)	(0.189)	(0.255)	(0.340)
*Childhood remoteness level*				
Small/medium city	0.0601	0.0932	0.0623	− 0.0757
	(0.0377)	(0.0850)	(0.124)	(0.137)
Rural/regional	0.121***	0.159**	0.263**	0.227*
	(0.0354)	(0.0799)	(0.114)	(0.133)
A mix	0.277**	0.471*	0.353	0.182
	(0.123)	(0.261)	(0.374)	(0.451)
Constant	2.122***		− 2.145***	0.463
	(0.0879)		(0.302)	(0.303)
N	3445	3421	3481	2018
R-squared/Pseudo R-squared	0.065	0.0177	0.0338	0.0519

***significant at 1% level ** significant at 5% level * significant at 10% level

Regression coefficients shown, standard error in parentheses. Base case is aged over 70, identifies as male, does not identify as Aboriginal and/or Torres Strait Islander, does not have a disability, speaks only English at home, highest level of education is high school completion (Year 12 certificate), employed full time, is in the middle income quintile, currently lives in NSW, lives in a major city, and grew up in a large/capital city

### Associations between PEBs, nature connection, and well-being

The models indicate a significant association (*p* < 0.01) between nature connection and PEBs across all four outcome variables when controlling for a range of socio-demographic variables (Table 3, Table S3 Supplementary for full model output). To put this finding into context, if we separate the CN-12 into quintiles and identify individuals who responded that they undertake at least one advocacy or other public sphere behaviour ‘sometimes’ or more frequently, we find that those in the highest CN-12 quintile are 81.7 times more likely to engage in any advocacy or other public sphere behaviour at least sometimes than those in the lowest CN-12 quintile. Those in the highest CN-12 quintile are 16.0 times more likely to report consumer-conscious behaviour at least sometimes than those in the lowest quintile. Finally, those in the highest CN-12 quintile are 3.7 times more likely to engage in conservation activities, and 1.8 times more likely to engage in gardening/lawncare activities, compared to those in the lowest CN-12 quintile (see Table S4 in Supplementary Information for full output).

**Table 3:** Regression coefficients for models assessing the relationship between the CN-12 and PEBs

Variables	Advocacy behaviours	Consumer-conscious behaviours	Conservation behaviours	Gardening and lawncare
CN-12	0.396***	0.898***	0.425***	0.198***
	(0.0105)	(0.0329)	(0.0463)	(0.0450)
Controls				

***significant at 1% level ** significant at 5% level * significant at 10% level

Regression coefficients shown, standard error in parentheses. Base case is aged over 70, identifies as male, does not identify as Aboriginal and/or Torres Strait Islander, does not have a disability, speaks only English at home, highest level of education is high school completion (Year 12 certificate), employed full time, is in the middle income quintile, currently lives in NSW, lives in a major city, and grew up in a large/capital city

These associations were also examined when using the INS and environmental visitation as measures for nature connection. Strong positive associations were found between all PEBs, except between the INS and gardening and lawncare, which did not have a clear pattern (Tables S5 and S6 Supplementary).

The OLS models indicated a significant relationship between all PEBs and life satisfaction (Table 4, *p* < 0.01). However, when controlling for CN-12 (i.e. including it in the model), only the advocacy behaviours variable remained significant (*p* < 0.05). See Table S7 Supplementary Information for full model details.

**Table 4:** Regression coefficients for models assessing the relationship between the PEBs and life satisfaction

Variables	PWI		PWI		PWI		PWI	
	Without CN-12	Controlling for CN-12	Without CN-12	Controlling for CN-12	Without CN-12	Controlling for CN-12	Without CN-12	Controlling for CN-12
Advocacy behaviours	0.285***	0.0754**						
	(0.0323)	(0.0379)						
Consumer-conscious behaviours								
Rarely			0.199*	− 0.0003				
			(0.113)	(0.112)				
Sometimes			0.242**	− 0.0863				
			(0.0963)	(0.0987)				
Often			0.474***	0.0067				
			(0.0985)	(0.105)				
Very often			0.714***	0.134				
			0.199*	− 0.000345				
Conservation behaviours					0.207***	0.0617		
					(0.0685)	(0.0675)		
Gardening and lawncare							0.199**	0.122
							(0.0776)	(0.0760)
Controls								

***significant at 1% level ** significant at 5% level * significant at 10% level

Regression coefficients shown, standard error in parentheses. Base case is aged over 70, identifies as male, does not identify as Aboriginal and/or Torres Strait Islander, does not have a disability, speaks only English at home, highest level of education is high school completion (Year 12 certificate), employed full time, is in the middle income quintile, currently lives in NSW, lives in a major city, and grew up in a large/capital city

## Limitations

Several limitations of this study should be acknowledged. First, the study relied on an online panel survey. Despite efforts to achieve diversity among Australian respondents, this approach inherently excludes individuals with lower literacy in written English and less familiarity with technology. While the overall sample size aimed to broadly reflect the Australian population, certain groups were under-represented. These included Indigenous Australians, individuals whose highest education level is high school, and people who speak a language other than English (see Table S1 in Supplementary Information). The sample opted for equal representation across states and to over-represent regional and rural populations so that conclusions could be drawn about all of these populations which would otherwise have been limited by a strictly population-representative design (which would have over-represented population centres of Sydney and Melbourne).

Second, the measure used to evaluate nature connectedness, the CN-12, may not fully capture the perspectives of non-Western worldviews regarding human–nature relationships. Different cultures have distinct ways of understanding and expressing connections with nature (Taylor 2018; Keaulana et al. 2021; Sedawi et al. 2021). Therefore, the CN-12 may not accurately reflect the ways that Indigenous Australians and other culturally diverse groups connect with non-human nature.

Third, while the advocacy and other public sphere behaviours scale comprehensively assessed a range of different behaviours, the other three measures—consumer-conscious behaviour, conservation activities, and gardening/lawncare—were single-item measures. In future, these constructs could be more comprehensively assessed through a scale examining various forms of these behaviours.

Finally, the variables for conservation activities and gardening/lawncare were compiled through a series of questions asking about activities conducted in a range of environments (for which protecting or caring for nature and engaging in gardening or lawncare activities were two). While this allowed for deeper analysis of the types of environments that people engage in these activities, the question also demanded a greater cognitive load (i.e. the amount of mental effort required to answer the question) for respondents (and thus may be downwardly biased). Individuals may have responded differently if asked these questions in general, rather than in relation to specific environments. Additionally, it should be noted that lawncare may not constitute a PEB. However, given that engagement in lawncare was incorporated into the same question as gardening, which we considered a ‘caring for nature’ activity, it was included in this analysis. It should also be noted that a programming error in administering the survey resulted in the gardening or lawncare activity being excluded from the original list of response options. While a supplementary follow-up survey sent to the initial respondents assessed engagement in gardening and lawncare across the three relevant environments, the response rate was lower for this supplementary survey (58.3% of initial respondents).

## Discussion

Our analysis firstly shows that there is a high level of variation in the types of PEBs undertaken by individuals in Australia, with consumer-conscious behaviours being the most frequently engaged in. When controlling for other life circumstances, we found that some population groups were more likely to engage in certain PEBs. Women were more likely to undertake all PEB types except gardening/lawncare. People who identify as non-binary, who have disability/ies or speak a language other than English at home were more likely to engage in advocacy behaviours. Our gender-related findings align with much of the previous research that people who identify as women typically have higher HNC, environmental concern and PEBs (Zelezny et al. 2000). However, there is variation in gender associations across studies suggesting an influence of gender-based cultural norms. For example, in China (Xiao and Hong 2010) and England (Martin et al. 2020; Table S6b) women engaged in more PEBs inside the home, while outside the home there were no differences in engagement across genders. A study with school children in Japan found that boys had a higher level of affective attitudes and willingness to conserve biodiversity (Soga et al. 2016). There are interesting intersections between gender and culture that are impacting experiences of nature connection.

We found that younger adults were more likely to engage in advocacy and conservation behaviours, older Australians were more likely to engage in consumer-conscious behaviours and gardening/lawncare. These age-related findings are somewhat surprising because previous research in the UK (Barrable and Booth 2022) and Australia (Dean et al. 2018; Meis-Harris et al. 2019; Selinske et al. 2023; Sollis et al. 2025) has found older people to have the highest, and young people the lowest, levels of nature connectedness. And previous research in the UK has found older people to be more likely to engage in pro-nature conservation behaviour (Richardson and Hamlin 2021). Despite nature connection being a strong predictor of PEBs across all PEB types in this research, we saw variation in the strength of that association across ages and PEB types. For younger people, the association between nature connection and the least frequently undertaken behaviour for this group—gardening/lawncare—was particularly strong. Therefore, while nature connection is generally lower for younger adults in Australia, other social or cultural factors may be motivating them to achieve the highest levels of advocacy and public sphere behaviours, and conservation actions in this study. Furthermore, nature connection may be a particularly strong motivator for younger people to engage in gardening and lawncare. The relationships between age, gender, HNC and PEBs appear to have nuances that warrant further exploration through additional research.

Differences in behaviour across the rural to urban spectrum were also detected: individuals who grew up in rural or regional areas of Australia were significantly more likely to engage in advocacy, consumer-conscious behaviours, and conservation behaviours (when controlling for other characteristics). Many other studies have observed that rural areas have higher nature connection which is correlated with high levels of PEB (Duron-Ramos et al. 2020; Anderson and Krettenauer 2021). A study in Bhutan which explored different types of behaviours found that rural residents had greater participation in personal gardening but lesser engagement in recycling, while urban residents practiced more recycling and water conservation (Yangchen et al. 2024). However, PEBs can differ widely based on the context of each situation leading to large variability across countries and cities. This was illustrated by Phuphisith et al. (2020), who found different PEB practices across Asian cities at different stages of development. Thus, the types of PEBs that individuals engage in will be context specific across the rural to urban gradient, but also across cities as the PEBs available and the cultural norms will emphasise engagement with different types of behaviours.

PEBs were most undertaken in one’s own yard or private green space. This corresponds with other research which has shown that private green spaces provide the easiest access for individuals who have them to interact with (Lin et al. 2017). Though these behaviours occur in private settings, demonstrating how private green spaces can be used in a sustainable way, such as wildlife-friendly gardens, can have flow-on effects and be considered a form of social advocacy (Jones et al. 2021). Promisingly, conservation behaviours were undertaken across all environments, albeit at a low frequency, including in built and programmed spaces such as zoos, highlighting the potential for landscape-scale conservation impacts.

Our analysis also indicated strong associations between nature connection and all types of PEBs examined. This result is not surprising and has commonly been observed in previous research (Martin et al. 2020; Whitburn et al. 2020; Liu et al. 2022). Like Gkargkavouzi et al. (2019), we found that nature connection predicted both private and public sphere behaviours, despite other research suggesting that individual characteristics are more likely to predict private sphere than public sphere behaviours (Alisat and Riemer 2015). Thus, our study contributes to the PEB literature in identifying how predictors of conservation behaviours may differ from predictors of more widely-studied PEBs such as recycling or energy and water conservation (van Eeden et al. 2024). Our findings highlight the critical role of nature connection tools (such as the CN-12 and INS), and our relationships with nature generally, for understanding pro-environmental behaviours within the broader literature.

Finally, a significant positive association was identified between all PEBs and life satisfaction. This could partially be due to the strong relationship between nature connection and well-being (e.g. Sollis et al. 2024). However, even when controlling for nature connection, advocacy and other public sphere behaviours remained significantly positively associated with life satisfaction, suggesting either that engaging in PEBs improves our well-being or that people with higher well-being are more likely to undertake PEBs. This finding is particularly important as the relationships between pro-environmental activism and mental well-being are unclear. For example, Whelan et al (2022) found a lower levels of mental well-being in people with higher levels of engagement in climate change issues. Schwartz et al. (2023) found collective climate action (but not individual climate action) to buffer the relationship between climate anxiety and major depressive symptoms. And when interviewing climate activists in Australia, Vercammen et al. (2024) found positive and negative impacts on well-being. Given these conflicting findings, as well as the relative dearth of studies examining this relationship, this finding makes an important contribution to the literature. Thus, the relationships and directionality of these three variables need to be investigated through further research.

### Leverage points for enhanced well-being and sustainability

Around the world, local, national and international policies recognise nature connection as a deep leverage point in transforming our relationship with the natural world and in helping to address the biodiversity crisis. For example, Protecting Victoria’s Environment-Biodiversity 2037, presents a 20-year strategy for the future of biodiversity including a central role for people connecting with nature and acting to protect it (Victoria State Government 2017). The associated Environmental Volunteering Plan has focused on engaging with and empowering young people, regional communities, First Nations and Traditional Owner groups, and pathways and communications to appeal to diverse audiences (Victorian State Government 2018). Our findings are consistent with advice that ‘policies that improve accessibility and support people to get out into natural environments are likely to play a key part in achieving health and sustainability objectives’ (Martin et al. 2020, p. 10).

Nationally, Australia’s Strategy for Nature, has at its #1 goal ‘to connect all Australians with nature’, recognising the links between connecting with nature and acting to protect it. Given that the 90% of the Australia population live in urban areas (Australian Bureau of Statistics 2024), the Strategy further underlines the critical role of nature in urban areas to ‘enrich cities’ and provide more opportunities for people to experience, connect with, and undertake nature activities (Commonwealth of Australia 2019). In response to such policies, governments have been investing in innovative and inclusive ways of connecting communities to nature and empowering them to engage in PEBs. For example, the Victoria Nature Festival (2019–2022) and Nature Festival South Australia (as Among It 2018–2019; 2020 onwards) directly emerged from state government biodiversity policies and include diverse content designed to engage across communities, in creative and accessible ways, for fun and meaningful nature experiences.

Although the extent to which such initiatives reach audiences with lower connection to nature is less clear, they still bring visibility to the crucial role of nature connection in our lives and for the planet, and as such, part of a broader societal shift. Further research could explore the impacts of such programs and identify pathways for a more nature-connective, environmentally-protective and life-satisfied population. Given that nature connection is a crucial leverage point for sustainability as part of broader systems shifts, policies and programs that connect people with nature in ways that benefits the environment and well-being should be part of business as usual for all governments, businesses, and communities across society.

## CONCLUSION

This paper has provided further evidence for the importance of nature connection as a leverage point for the well-being of people and planet. Our analysis highlights a strong association between nature connection and all types of PEBs we examined, despite variability in the types of behaviours engaged undertaken. We found that certain groups are more likely to engage in PEBs: those identifying as women, Indigenous Australians; people with disability/ies; those who speak a language other than English at home; and people who grew up in rural or regional areas. Further research could examine the drivers for these population groups to engage in PEBs to better understand how these behaviours could be enhanced at a whole-of-population level. Importantly, we found initial evidence of a link between life satisfaction and PEBs which should be explored further.

Public policies which recognise the co-benefits of nature connecting activities for well-being and PEBs are crucial for sustainability. Pursuing policies and programs that connect people with nature can have positive, reinforcing benefits, helping to enhance both the well-being of individuals and the health of the planet.

## Supplementary Information

Below is the link to the electronic supplementary material.

Supplementary file1 (PDF 669 KB)

## Footnotes

1 The different options for environments were: a forest; a grassland/savanna/open woodland; a desert/dry scrubland; a lake, river or other inland waterway; a wetland, marsh, or estuary; an agricultural area; the beach or coastal areas; on or in the ocean water; a zoo or wildlife park; a botanic garden or urban park with grassy lawns and trees; a community garden; and your own garden or yard at home.

2 The other options for activities were: rest and recover, disconnect from daily life; socialise; accompany children to activities; engage in physical activities; engage in motorised leisure activities; experience a sense of peace, tranquillity or awe; engage in cultural activities; enjoy nature; have a picnic or BBQ; Walk your pet; Pass through to reach your destination; look up at the moon or stars in the sky; go hunting; go fishing; learn about the plants, animals or environment; and other.

3 Lawncare may not constitute a PEB. It was included in this analysis due to its amalgamation with the activity of gardening. The shortcomings of this will be discussed in the Limitations section. A programming error resulted in the gardening or lawncare activity being excluded from the list of response options. A supplementary follow-up survey sent to the initial respondents assessed engagement in gardening and lawncare across three relevant environments: agricultural area, community garden, and your own garden or yard at home. The response rate for the supplementary survey was 58.3% of initial respondents.
