# Supplementary material for: Protecting nature in diverse ways: The socio-demographic spread of benefits from connecting with nature
Source: Ambio. 2025 Aug 21;55(2):312–26. doi: 10.1007/s13280-025-02233-6 (PMC12779869; doi:10.1007/s13280-025-02233-6)
Supplement: Supplementary file 1 — Supplementary file1 (PDF 669 KB) [file 13280_2025_2233_MOESM1_ESM.pdf]

Protecting nature in diverse ways: The socio-demographic spread of benefits from connecting with nature

Kate Sollis[1], Lily van Eeden[2], Usitha Rajeevan[3], Kate Lee[4], Brenda Lin[5], Lucy Keniger[6], Pauline Marsh[7*], & Emily Flies[8*]

*Senior authors

1 Geography, Planning and Spatial Sciences, University of Tasmania, Sandy Bay Campus, Hobart, TAS, AU 7001. Kate.Sollis@utas.edu.au. ORCID ID: 0000-0002-0636-8805

2 RMIT University STEM College, Applied Chemistry and Environmental Sciences Melbourne, VIC, AU 3000. lily.van.eeden@rmit.edu.au. ORCID ID: 0000-0002-0456-9670

3 Geography, Planning and Spatial Sciences, University of Tasmania, Sandy Bay Campus, Hobart, TAS, AU 7001. usitha.rajeevan@utas.edu.au. ORCID ID: 0009-0000-0925-1568

4 Arthur Rylah Institute for Environmental Research, Department of Energy, Environment, and Climate Action, Government of Victoria, Heidelberg, Victoria; School of Agriculture, Food and Ecosystem Sciences, University of Melbourne, Parkville, Victoria; BehaviourWorks Australia, Monash University, Clayton, Victoria. kate.lee@delwp.vic.gov.au. ORCID ID: 0000-0002-8329-079X

5 CSIRO, Land & Water Ecosystem Sciences Precinct 41 Boggo Road Dutton Park, QLD, AU 4102. Brenda.Lin@csiro.au. ORCID ID: 0000-0002-6011-9172

6 Geography, Planning and Spatial Sciences, University of Tasmania, Sandy Bay Campus, Hobart, TAS, AU 7001. lucy@keniger.com.

7 Wicking Dementia Research and Education Centre, University of Tasmania, Sandy Bay Campus, Hobart, TAS, AU 7001. pauline.marsh@utas.edu.au. ORCID ID: 0000-0002-4371-2628

8 Geography, Planning and Spatial Sciences, University of Tasmania, Sandy Bay Campus, Hobart, TAS, AU 7001. emily.flies@utas.edu.au. ORCID ID: 0000-0002-1013-0330

Corresponding author: Kate Sollis – Kate.Sollis@utas.edu.au

Supplementary information

Table S1 Sample characteristics

Variable	n (%)	Percentage	National benchmark (2021 Census)
Age			
18-30 years	832	20.8%	21.6%
31-50 years	1,289	32.1%	35.0%
51-70 years	1,334	33.3%	29.2%
>70 years	551	13.8%	14.3%
Gender[1]			
Male	1,913	47.8%	49.3%
Female	2,074	51.9%	50.7%
Non-binary	12	0.3%	N/A
Indigenous status			
Identifies as Aboriginal and/or Torres Strait Islander	79	2.0%	3.4%
Non-Indigenous	3,883	98.0%	96.6%
Disability			
Has disability/ies	363	9.1%	N/A
No disability/ies	3,643	90.9%	N/A
Linguistic diversity			
Speaks a language other than English at home	365	9.1%	22.3%
Speaks only English	3,641	90.9%	77.7%
Educational status			
Has not completed high school (Year 12)	353	8.8%	8.1%
High school completion (Year 12)	561	14%	33.4%
Certificate/Diploma	1,183	29.5%	31.5%
Undergraduate	1,052	26.3%	19.6%
Postgraduate	857	21.4%	7.4%
Employment status			
Full-time	1,716	42.9%	38.2%
Part-time	452	11.3%	21.3%
Casually employed	243	6.1%	N/A
Self-employed	258	6.4%	N/A
Home duties/volunteer work	208	5.2%	N/A
Retired	929	23.2%	N/A
Not working or studying	133	3.3%	N/A
Student only	65	1.6%	N/A
Income quintile			
Lowest income quintile	573	16.2%	20%
Second-lowest income quintile	813	22.9%	20%
Middle income quintile	527	14.9%	20%
Second-highest income quintile	1,015	28.6%	20%
Highest income quintile	616	17.4%	20%
State of current location			
NSW	537	13.4%	31.8%
Victoria	533	13.3%	25.6%
Queensland	534	13.3%	20.3%
South Australia	534	13.3%	7.0%
Western Australia	535	13.4%	10.5%

[1] The national benchmark figures for gender are based on a question asking the respondent's sex, for which only male and female are options.

Tasmania	520	13.0%	2.2%
Australian Capital Territory	507	12.7%	1.8%
Northern Territory	306	7.6%	0.9%
Current remoteness level			
Major city	2,155	53.8%	72.2%
Regional	1,699	42.4%	26.0%
Remote	152	3.8%	1.9%
Remoteness level of childhood environment			
Large/capital city	1,791	44.7%	N/A
Small/medium city	913	22.8%	N/A
Rural/regional area	1,249	31.2%	N/A
A mix	53	1.3%	N/A

Table S2 Variance Inflation Factor analysis

	VIF	1/VIF
CN-12	1.05	0.954889
Age		
Aged 18-30 years	3.89	0.256941
Aged 31-50 years	4.58	0.218183
Aged 51-70 years	3.26	0.306934
**Gender**		
Identifies as female	1.11	0.901776
Non-binary	1.02	0.978291
Identifies as Aboriginal and/or Torres Strait Islander	1.04	0.95992
Has disability/ies	1.1	0.906385
Speaks language other than English at home	1.07	0.931236
**Highest level of education**		
Has not completed high school (Year 12)	1.56	0.641489
Certificate/Diploma	2.32	0.43038
Undergraduate	2.26	0.442524
Postgraduate	2.22	0.451201
**Employment status**		
Part-time	1.22	0.818458
Casual	1.18	0.846713
Self-employed	1.14	0.875033
Engaged in home duties/volunteer work	1.17	0.854312
Retired	2.56	0.390916
Not working/studying	1.19	0.842115
Student only	1.06	0.939236
**Personal income quintile**		
Lowest income quintile	2.01	0.496481
2nd-lowest income quintile	2.04	0.490452
4th-highest income quintile	2.13	0.468761
Highest income quintile	1.96	0.509817
Standardised IRSAD score	1.75	0.571068
**State/territory**		
Victoria	1.73	0.577982
Queensland	1.77	0.564902
South Australia	1.82	0.549077
Western Australia	1.79	0.55762
Tasmania	2.03	0.493715
Australian Capital Territory	1.99	0.502866
Northern Territory	1.8	0.556858
**Current remoteness level**		
Regional	2.2	0.453516
Remote	1.39	0.719331
**Childhood remoteness level**		
Small/medium city	1.32	0.757716
Rural/regional	1.44	0.692217
A mix	1.03	0.970012
**Mean VIF**	1.79	

Note: Base case is aged over 70, identifies as male, does not identify as Aboriginal and/or Torres Strait Islander, does not have a disability, speaks only English at home, highest level of education is high school completion (Year 12 certificate), employed full time, is in the middle income quintile, currently lives in NSW, lives in a major city, and grew up in a large/capital city.

Table S3 Full model output for regression models assessing relationship between CN-12 and PEBs

VARIABLES	(1)<br>Advocacy behaviours	(2)<br>Consumer-conscious behaviours	(3)<br>Conservation behaviours	(4)<br>Gardening and lawncare
CN-12	0.396***	0.898***	0.425***	0.198***
	(0.0105)	(0.0329)	(0.0463)	(0.0450)
**Age**				
18-30 years	0.123**	-0.232	1.058***	-0.873***
	(0.0554)	(0.152)	(0.237)	(0.267)
31-50 years	0.0218	-0.437***	0.711***	-0.424**
	(0.0524)	(0.144)	(0.229)	(0.205)
51-70 years	-0.0805*	-0.324***	0.292	-0.0645
	(0.0445)	(0.122)	(0.202)	(0.169)
**Gender**				
Identifies as female	0.0352	0.355***	0.126	0.0204
	(0.0243)	(0.0667)	(0.0949)	(0.108)
Non-binary	0.496**	1.400**	1.233*	
	(0.217)	(0.598)	(0.673)	
Identifies as Aboriginal and/or Torres Strait Islander	0.258***	0.244	0.329	0.859
	(0.0828)	(0.227)	(0.282)	(0.575)
Has disability/ies	0.147***	0.267**	0.224	-0.177
	(0.0432)	(0.120)	(0.165)	(0.174)
Speaks language other than English at home	0.0961**	-0.0135	-0.0115	-0.0870
	(0.0410)	(0.111)	(0.152)	(0.186)
**Highest level of education**				
Has not completed high school (Year 12)	-0.0987*	-0.276*	-0.486**	-0.401*
	(0.0526)	(0.145)	(0.234)	(0.218)
Certificate/Diploma	0.0153	0.0750	-0.0118	0.0289
	(0.0383)	(0.105)	(0.149)	(0.175)
Undergraduate	0.157***	0.206*	0.0727	0.0884
	(0.0392)	(0.107)	(0.149)	(0.182)
Postgraduate	0.223***	0.433***	0.0155	-0.0443
	(0.0419)	(0.115)	(0.163)	(0.188)
**Employment status**				
Part-time	0.00312	-0.0380	-0.170	0.189
	(0.0397)	(0.109)	(0.151)	(0.179)
Casual	0.00877	-0.0207	0.0535	0.166
	(0.0532)	(0.146)	(0.196)	(0.264)
Self-employed	-0.0601	0.0794	-0.0265	0.107
	(0.0506)	(0.141)	(0.198)	(0.215)
Engaged in home duties/volunteer work	-0.0886	-0.154	-0.0768	0.804***
	(0.0576)	(0.158)	(0.216)	(0.277)
Retired	-0.0940**	-0.132	-0.0942	0.390**
	(0.0447)	(0.121)	(0.187)	(0.180)
Not working/studying	-0.0883	-0.186	-0.301	-0.177
	(0.0734)	(0.203)	(0.294)	(0.305)
Student only	0.206**	0.544**	0.610**	0.249
	(0.0967)	(0.258)	(0.309)	(0.584)
**Personal income quintile**				
Lowest income quintile	-0.0246	-0.153	-0.260	-0.211
	(0.0449)	(0.123)	(0.174)	(0.196)
2nd-lowest income quintile	0.000530	0.00110	-0.200	-0.215
	(0.0393)	(0.107)	(0.149)	(0.175)
4th-highest income quintile	-0.0520	0.150	-0.167	0.0936

	(0.0372)	(0.100)	(0.138)	(0.171)
Highest income quintile	-0.130***	0.104	-0.448***	-0.0403
	(0.0424)	(0.115)	(0.164)	(0.191)
Standardised IRSAD score	0.0289*	0.0299	-0.0137	-0.170***
	(0.0153)	(0.0418)	(0.0594)	(0.0658)
**State/territory**				
Victoria	-0.0214	-0.0415	0.0209	0.231
	(0.0452)	(0.125)	(0.174)	(0.178)
Queensland	-0.0520	-0.163	-0.242	0.0382
	(0.0451)	(0.125)	(0.183)	(0.177)
South Australia	-0.0434	0.252**	-0.0732	0.438**
	(0.0458)	(0.126)	(0.180)	(0.195)
Western Australia	-0.0123	0.188	0.126	0.0818
	(0.0455)	(0.125)	(0.176)	(0.183)
Tasmania	-0.00408	0.192	-0.112	0.120
	(0.0487)	(0.134)	(0.189)	(0.229)
Australian Capital Territory	0.0105	0.416***	0.0550	0.488**
	(0.0487)	(0.133)	(0.190)	(0.211)
Northern Territory	0.0893	0.0546	-0.0692	0.172
	(0.0574)	(0.157)	(0.220)	(0.290)
**Current remoteness level**				
Regional	-0.0154	0.122	-0.00870	0.505***
	(0.0346)	(0.0946)	(0.136)	(0.151)
Remote	-0.132*	-0.0623	0.164	0.395
	(0.0701)	(0.196)	(0.258)	(0.343)
**Childhood remoteness level**				
Small/medium city	0.0362	0.0481	0.0438	-0.0978
	(0.0317)	(0.0865)	(0.125)	(0.138)
Rural/regional	0.0451	-0.00838	0.197*	0.204
	(0.0298)	(0.0818)	(0.116)	(0.134)
A mix	0.191*	0.339	0.299	0.116
	(0.103)	(0.261)	(0.377)	(0.452)
Constant	0.185**		-4.287***	-0.497
	(0.0897)		(0.390)	(0.373)
Observations	3,445	3,421	3,481	2,018
R-squared/Pseudo R-squared	0.342	0.1000	0.0618	0.0597

Note: *** significant at 1% level ** significant at 5% level * significant at 10% level. Regression coefficients shown, standard error in parentheses. Base case is aged over 70, identifies as male, does not identify as Aboriginal and/or Torres Strait Islander, does not have a disability, speaks only English at home, highest level of education is high school completion (Year 12 certificate), employed full time, is in the middle income quintile, currently lives in NSW, lives in a major city, and grew up in a large/capital city.

Table S4 Logit models identifying odds ratios for relationships between PEBs and nature connection (CN-12), and socio-demographic variables

VARIABLES	(1) Any advocacy or other public sphere behaviour at least sometimes	(2) Consumer conscious behaviour at least sometimes	(3) Conservation behaviours	(4) Gardening and lawncare
**CN-12 quintiles**				
2nd lowest quintile	3.917***	2.490***	1.536**	1.034
	(0.578)	(0.302)	(0.259)	(0.161)
Middle quintile	6.956***	4.477***	2.120***	1.423**
	(1.133)	(0.576)	(0.339)	(0.220)
2nd highest quintile	13.17***	7.410***	2.634***	1.410**
	(2.571)	(1.053)	(0.410)	(0.218)
Highest quintile	81.70***	16.00***	3.677***	1.814***
	(37.56)	(3.115)	(0.585)	(0.300)
**Age**				
18-30 years	1.707*	1.012	2.844***	0.415***
	(0.472)	(0.222)	(0.672)	(0.111)
31-50 years	1.206	0.820	2.027***	0.649**
	(0.310)	(0.170)	(0.464)	(0.133)
51-70 years	0.748	0.855	1.328	0.925
	(0.161)	(0.151)	(0.268)	(0.156)
**Gender**				
Identifies as female	1.267*	1.457***	1.135	1.020
	(0.153)	(0.139)	(0.108)	(0.110)
Non-binary	-	4.697	3.566*	-
		(5.137)	(2.397)	
Identifies as Aboriginal and/or Torres Strait Islander	1.078	1.168	1.373	2.296
	(0.517)	(0.399)	(0.386)	(1.323)
Has disability/ies	1.232	1.260	1.253	0.832
	(0.270)	(0.223)	(0.206)	(0.145)
Speaks language other than English at home	1.050	1.039	0.995	0.915
	(0.236)	(0.174)	(0.151)	(0.170)
**Highest level of education**				
Has not completed high school (Year 12)	0.870	0.723*	0.614**	0.666*
	(0.197)	(0.138)	(0.143)	(0.145)
Certificate/Diploma	1.229	1.074	0.986	1.033
	(0.212)	(0.153)	(0.146)	(0.181)
Undergraduate	2.202***	1.365**	1.081	1.112
	(0.420)	(0.203)	(0.161)	(0.202)
Postgraduate	1.961***	1.951***	1.016	0.964
	(0.397)	(0.325)	(0.166)	(0.181)
**Employment status**				
Part-time	1.292	1.122	0.845	1.211
	(0.267)	(0.179)	(0.128)	(0.218)
Casual	1.858**	1.239	1.053	1.197
	(0.545)	(0.263)	(0.206)	(0.317)
Self-employed	1.062	1.002	0.977	1.104
	(0.266)	(0.197)	(0.194)	(0.237)
Engaged in home duties/volunteer work	1.046	0.831	0.918	2.185***
	(0.305)	(0.184)	(0.198)	(0.604)
Retired	1.305	1.226	0.909	1.480**
	(0.281)	(0.217)	(0.169)	(0.267)
Not working/studying	0.800	0.797	0.727	0.838

	(0.271)	(0.221)	(0.213)	(0.256)
Student only	10.39**	4.853***	1.898**	1.295
	(10.75)	(2.657)	(0.587)	(0.760)
**Personal income quintile**				
Lowest income quintile	0.636**	0.822	0.778	0.815
	(0.144)	(0.145)	(0.136)	(0.160)
2nd-lowest income quintile	0.747	0.859	0.825	0.818
	(0.152)	(0.132)	(0.123)	(0.143)
4th-highest income quintile	0.794	1.068	0.855	1.118
	(0.155)	(0.156)	(0.118)	(0.192)
Highest income quintile	0.766	1.137	0.640***	0.972
	(0.166)	(0.190)	(0.105)	(0.185)
Standardised IRSAD score	1.165**	1.041	0.985	0.843***
	(0.0869)	(0.0619)	(0.0584)	(0.0555)
**State/territory**				
Victoria	0.755	1.024	1.022	1.248
	(0.158)	(0.173)	(0.178)	(0.223)
Queensland	1.106	0.909	0.780	1.022
	(0.239)	(0.151)	(0.143)	(0.181)
South Australia	1.347	1.583***	0.933	1.554**
	(0.299)	(0.280)	(0.168)	(0.303)
Western Australia	1.090	1.600***	1.132	1.073
	(0.234)	(0.282)	(0.199)	(0.197)
Tasmania	1.135	1.290	0.889	1.115
	(0.274)	(0.246)	(0.168)	(0.256)
Australian Capital Territory	1.811**	1.715***	1.050	1.627**
	(0.455)	(0.328)	(0.200)	(0.343)
Northern Territory	1.418	1.139	0.927	1.169
	(0.454)	(0.265)	(0.203)	(0.339)
**Current remoteness level**				
Regional	1.458**	1.321**	0.990	1.656***
	(0.243)	(0.177)	(0.134)	(0.251)
Remote	1.222	0.896	1.171	1.469
	(0.448)	(0.250)	(0.302)	(0.503)
**Childhood remoteness level**				
Small/medium city	0.951	1.116	1.045	0.914
	(0.148)	(0.139)	(0.131)	(0.126)
Rural/regional	1.069	1.027	1.223*	1.234
	(0.161)	(0.121)	(0.142)	(0.165)
A mix	3.435	2.928*	1.348	1.155
	(2.788)	(1.669)	(0.509)	(0.523)
Constant	0.789	0.522**	0.0669***	1.338
	(0.284)	(0.152)	(0.0217)	(0.422)
Observations	3,435	3,421	3,481	2,018
Pseudo R-squared	0.224	0.152	0.0596	0.0591

Note: *** significant at 1% level ** significant at 5% level * significant at 10% level. Odds ratios shown, standard error in parentheses. Base case is in the lowest CN-12 quintile, is aged over 70, identifies as male, does not identify as Aboriginal and/or Torres Strait Islander, does not have a disability, speaks only English at home, highest level of education is high school completion (Year 12 certificate), employed full time, is in the middle income quintile, currently lives in NSW, lives in a major city, and grew up in a large/capital city.

Table S5 OLS and logit models identifying relationship between different pro-environmental variables with nature connection (INS)

VARIABLES	(1)<br>Advocacy behaviours	(2)<br>Consumer-conscious behaviours	(3)<br>Conservation behaviours	(4)<br>Gardening and lawncare
**INS**				
A	-0.584***	-1.600***	-1.168***	-0.275
	(0.0662)	(0.179)	(0.357)	(0.249)
B	-0.518***	-1.011***	-0.749***	-0.516***
	(0.0469)	(0.119)	(0.198)	(0.189)
C	-0.281***	-0.504***	-0.349***	-0.136
	(0.0359)	(0.0894)	(0.135)	(0.148)
E	0.275***	0.622***	0.359***	0.179
	(0.0369)	(0.0931)	(0.124)	(0.153)
F	0.445***	0.594***	0.241	0.247
	(0.0477)	(0.121)	(0.165)	(0.190)
G	0.720***	1.171***	0.412**	-0.0780
	(0.0490)	(0.128)	(0.163)	(0.187)
**Age**				
18-30 years	0.167***	-0.190	1.075***	-0.870***
	(0.0599)	(0.151)	(0.235)	(0.268)
31-50 years	0.0646	-0.366**	0.738***	-0.386*
	(0.0566)	(0.143)	(0.228)	(0.206)
51-70 years	-0.0470	-0.252**	0.339*	-0.0506
	(0.0479)	(0.120)	(0.201)	(0.169)
**Gender**				
Identifies as female	0.0894***	0.455***	0.183*	0.0487
	(0.0261)	(0.0661)	(0.0944)	(0.107)
Non-binary	0.381	1.001*	1.040	
	(0.234)	(0.583)	(0.677)	
Identifies as Aboriginal and/or Torres Strait Islander	0.234***	0.268	0.360	0.802
	(0.0895)	(0.224)	(0.281)	(0.575)
Has disability/ies	0.161***	0.300**	0.270*	-0.158
	(0.0465)	(0.119)	(0.164)	(0.174)
Speaks language other than English at home	0.0909**	-0.0154	0.00156	-0.0754
	(0.0442)	(0.111)	(0.152)	(0.186)
**Highest level of education**				
Has not completed high school (Year 12)	-0.110*	-0.292**	-0.482**	-0.417*
	(0.0567)	(0.144)	(0.233)	(0.218)
Certificate/Diploma	0.0226	0.106	0.00270	0.0238
	(0.0413)	(0.104)	(0.148)	(0.175)
Undergraduate	0.209***	0.310***	0.117	0.101
	(0.0423)	(0.106)	(0.149)	(0.182)
Postgraduate	0.269***	0.535***	0.0659	-0.0209
	(0.0452)	(0.114)	(0.163)	(0.188)
**Employment status**				
Part-time	0.0439	0.0584	-0.133	0.211
	(0.0428)	(0.108)	(0.150)	(0.179)
Casual	0.0463	0.0387	0.0759	0.177
	(0.0574)	(0.144)	(0.195)	(0.265)
Self-employed	-0.0711	0.0592	-0.0324	0.0989
	(0.0546)	(0.140)	(0.198)	(0.214)
Engaged in home duties/volunteer work	-0.0548	-0.0759	-0.0264	0.806***
	(0.0620)	(0.155)	(0.215)	(0.275)

Retired	-0.0410	-0.0317	-0.0756	0.418**
	(0.0482)	(0.121)	(0.185)	(0.180)
Not working/studying	-0.0728	-0.0990	-0.276	-0.138
	(0.0791)	(0.199)	(0.293)	(0.306)
Student only	0.247**	0.511**	0.593*	0.237
	(0.104)	(0.258)	(0.307)	(0.585)
**Personal income quintile**				
Lowest income quintile	-0.0644	-0.176	-0.278	-0.210
	(0.0484)	(0.122)	(0.174)	(0.196)
2nd-lowest income quintile	-0.0102	-0.000177	-0.219	-0.218
	(0.0424)	(0.106)	(0.149)	(0.175)
4th-highest income quintile	-0.0501	0.132	-0.181	0.0903
	(0.0401)	(0.0999)	(0.137)	(0.171)
Highest income quintile	-0.171***	0.00283	-0.501***	-0.0514
	(0.0457)	(0.114)	(0.163)	(0.191)
Standardised IRSAD score	0.0281*	0.0367	-0.0106	-0.160**
	(0.0164)	(0.0413)	(0.0591)	(0.0656)
**State/territory**				
Victoria	0.00525	-0.0231	0.0345	0.251
	(0.0487)	(0.125)	(0.174)	(0.179)
Queensland	-0.0418	-0.150	-0.227	0.0506
	(0.0487)	(0.124)	(0.182)	(0.177)
South Australia	-0.0264	0.268**	-0.0541	0.467**
	(0.0493)	(0.125)	(0.179)	(0.195)
Western Australia	0.0390	0.294**	0.168	0.0989
	(0.0490)	(0.124)	(0.175)	(0.184)
Tasmania	0.0172	0.187	-0.0845	0.132
	(0.0524)	(0.133)	(0.189)	(0.229)
Australian Capital Territory	0.0715	0.475***	0.0996	0.489**
	(0.0526)	(0.133)	(0.190)	(0.211)
Northern Territory	0.156**	0.160	-0.00299	0.208
	(0.0618)	(0.156)	(0.220)	(0.289)
**Current remoteness level**				
Regional	0.0116	0.177*	0.00958	0.508***
	(0.0373)	(0.0939)	(0.135)	(0.151)
Remote	-0.132*	-0.0538	0.166	0.412
	(0.0755)	(0.192)	(0.258)	(0.342)
**Childhood remoteness level**				
Small/medium city	0.0398	0.0611	0.0437	-0.0862
	(0.0341)	(0.0858)	(0.125)	(0.138)
Rural/regional	0.0679**	0.0727	0.206*	0.227*
	(0.0321)	(0.0811)	(0.116)	(0.134)
A mix	0.249**	0.401	0.335	0.167
	(0.111)	(0.262)	(0.373)	(0.454)
Constant	2.123***		-2.068***	0.512
	(0.0815)		(0.310)	(0.313)
Observations	3,445	3,421	3,481	2,018
R-squared/Pseudo R-squared	0.238	0.0579	0.0540	0.0583

Note: *** significant at 1% level ** significant at 5% level * significant at 10% level. Regression coefficients shown, standard error in parentheses. Base case responds D to the INS, is aged over 70, identifies as male, does not identify as Aboriginal and/or Torres Strait Islander, does not have a disability, speaks only English at home, highest level of education is high school completion (Year 12 certificate), employed full time, is in the middle income quintile, currently lives in NSW, lives in a major city, and grew up in a large/capital city.

Table S6 OLS and logit models identifying relationship between different PEB variables dimensions with environmental visitation

VARIABLES	(1)<br>Advocacy behaviours	(2)<br>Consumer-conscious behaviours	(3)<br>Conservation behaviours	(4)<br>Gardening and lawncare
**Time in nature**				
Less than once a year	0.0675	0.0647	0.404	0.213
	(0.139)	(0.351)	(1.138)	(0.442)
At least once a year	0.375***	0.539	2.055*	0.443
	(0.139)	(0.351)	(1.052)	(0.454)
At least twice a year	0.407***	0.991***	1.474	0.790**
	(0.121)	(0.310)	(1.031)	(0.396)
At least once a month	0.598***	1.300***	1.637	0.700*
	(0.119)	(0.305)	(1.025)	(0.387)
At least once a fortnight	0.775***	1.631***	1.940*	0.858**
	(0.122)	(0.312)	(1.028)	(0.402)
At least once a week	0.704***	1.633***	2.072**	0.976**
	(0.119)	(0.305)	(1.022)	(0.389)
A couple times a week	0.835***	1.794***	2.080**	0.913**
	(0.117)	(0.301)	(1.021)	(0.382)
Every day	1.012***	2.148***	2.267**	1.157***
	(0.116)	(0.301)	(1.020)	(0.381)
**Age**				
18-30 years	-0.00553	-0.494***	0.883***	-0.946***
	(0.0634)	(0.150)	(0.235)	(0.267)
31-50 years	-0.0162	-0.502***	0.641***	-0.445**
	(0.0600)	(0.142)	(0.228)	(0.206)
51-70 years	-0.0888*	-0.325***	0.275	-0.0966
	(0.0510)	(0.120)	(0.201)	(0.170)
**Gender**				
Identifies as female	0.0758***	0.398***	0.154	0.0291
	(0.0278)	(0.0662)	(0.0946)	(0.108)
Non-binary	0.467*	1.097*	1.146*	
	(0.248)	(0.575)	(0.684)	
Identifies as Aboriginal and/or Torres Strait Islander	0.309***	0.355	0.394	0.827
	(0.0949)	(0.227)	(0.279)	(0.578)
Has disability/ies	0.203***	0.362***	0.294*	-0.127
	(0.0494)	(0.119)	(0.164)	(0.174)
Speaks language other than English at home	0.178***	0.152	0.0636	-0.0633
	(0.0469)	(0.110)	(0.151)	(0.186)
**Highest level of education**				
Has not completed high school (Year 12)	-0.0831	-0.221	-0.470**	-0.391*
	(0.0603)	(0.143)	(0.233)	(0.220)
Certificate/Diploma	0.0565	0.140	0.0160	0.0123
	(0.0438)	(0.104)	(0.148)	(0.176)
Undergraduate	0.196***	0.238**	0.0922	0.0366
	(0.0450)	(0.106)	(0.148)	(0.183)
Postgraduate	0.265***	0.467***	0.0415	-0.106
	(0.0480)	(0.114)	(0.162)	(0.189)
**Employment status**				
Part-time	0.0227	0.0151	-0.165	0.171
	(0.0455)	(0.107)	(0.150)	(0.180)
Casual	-0.0231	-0.0847	0.00615	0.0995
	(0.0610)	(0.144)	(0.194)	(0.265)
Self-employed	-0.0701	0.0592	-0.0358	0.0764
	(0.0581)	(0.139)	(0.198)	(0.215)

Engaged in home duties/volunteer work	-0.115*	-0.186	-0.100	0.730***
	(0.0661)	(0.156)	(0.216)	(0.277)
Retired	-0.110**	-0.150	-0.152	0.356*
	(0.0515)	(0.121)	(0.186)	(0.182)
Not working/studying	-0.0949	-0.163	-0.290	-0.193
	(0.0841)	(0.201)	(0.293)	(0.306)
Student only	0.269**	0.574**	0.643**	0.256
	(0.111)	(0.258)	(0.308)	(0.583)
**Personal income quintile**				
Lowest income quintile	0.0315	-0.0272	-0.167	-0.179
	(0.0514)	(0.121)	(0.173)	(0.197)
2nd-lowest income quintile	0.0199	0.0506	-0.167	-0.204
	(0.0450)	(0.106)	(0.148)	(0.176)
4th-highest income quintile	-0.0575	0.124	-0.167	0.100
	(0.0425)	(0.0995)	(0.137)	(0.172)
Highest income quintile	-0.200***	-0.0448	-0.489***	-0.0381
	(0.0486)	(0.114)	(0.163)	(0.191)
Standardised IRSAD score	0.0306*	0.0383	-0.0141	-0.174***
	(0.0175)	(0.0413)	(0.0590)	(0.0661)
**State/territory**				
Victoria	-0.00260	-0.00575	0.0458	0.221
	(0.0519)	(0.124)	(0.174)	(0.179)
Queensland	-0.0452	-0.139	-0.220	0.0442
	(0.0517)	(0.124)	(0.182)	(0.178)
South Australia	-0.0414	0.241*	-0.0713	0.423**
	(0.0524)	(0.125)	(0.179)	(0.196)
Western Australia	-0.0256	0.157	0.107	0.0788
	(0.0521)	(0.123)	(0.175)	(0.184)
Tasmania	-0.0201	0.144	-0.108	0.133
	(0.0558)	(0.133)	(0.188)	(0.231)
Australian Capital Territory	-0.0319	0.293**	0.0105	0.446**
	(0.0558)	(0.133)	(0.189)	(0.211)
Northern Territory	0.144**	0.140	0.00229	0.190
	(0.0658)	(0.156)	(0.219)	(0.290)
**Current remoteness level**				
Regional	-0.0432	0.0784	-0.0503	0.459***
	(0.0398)	(0.0939)	(0.136)	(0.153)
Remote	-0.150*	-0.0920	0.141	0.351
	(0.0803)	(0.190)	(0.258)	(0.344)
**Childhood remoteness level**				
Small/medium city	0.0610*	0.0925	0.0543	-0.0892
	(0.0363)	(0.0857)	(0.124)	(0.139)
Rural/regional	0.0825**	0.0838	0.215*	0.206
	(0.0341)	(0.0805)	(0.115)	(0.134)
A mix	0.248**	0.426	0.356	0.118
	(0.118)	(0.264)	(0.374)	(0.453)
Constant	1.528***		-3.911***	-0.240
	(0.137)		(1.053)	(0.456)
Observations	3,445	3,421	3,481	2,018
R-squared/Pseudo R-squared	0.141	0.0404	0.0487	0.0612

Note: *** significant at 1% level ** significant at 5% level * significant at 10% level. Regression coefficients shown, standard error in parentheses. Base case never spends time in nature, is aged over 70, identifies as male, does not identify as Aboriginal and/or Torres Strait Islander, does not have a disability, speaks only English at home, highest level of education is high school completion (Year 12 certificate), employed full time, is in the middle income quintile, currently lives in NSW, lives in a major city, and grew up in a large/capital city.

Table S7 OLS models identifying relationship between PEBs and life satisfaction

	PWI		PWI		PWI		PWI	
VARIABLES	*Without CN-12*	*Controlling for CN-12*	*Without CN-12*	*Controlling for CN-12*	*Without CN-12*	*Controlling for CN-12*	*Without CN-12*	*Controlling for CN-12*
Advocacy behaviours	0.285***	0.0754**						
	(0.0323)	(0.0379)						
**Consumer-conscious behaviours**								
Rarely			0.199*	-0.000345				
			(0.113)	(0.112)				
Sometimes			0.242**	-0.0863				
			(0.0963)	(0.0987)				
Often			0.474***	0.00673				
			(0.0985)	(0.105)				
Very often			0.714***	0.134				
			(0.116)	(0.125)				
Conservation behaviours					0.207***	0.0617		
					(0.0685)	(0.0675)		
Gardening and lawncare							0.199**	0.122
							(0.0776)	(0.0760)
CN-12		0.280***		0.300***		0.321***		0.318***
		(0.0276)		(0.0262)		(0.0234)		(0.0309)
**Age**								
18-30 years	-0.656***	-0.580***	-0.627***	-0.584***	-0.674***	-0.577***	-0.417**	-0.340*
	(0.124)	(0.123)	(0.125)	(0.123)	(0.126)	(0.123)	(0.190)	(0.185)
31-50 years	-0.781***	-0.756***	-0.743***	-0.750***	-0.801***	-0.758***	-0.771***	-0.754***
	(0.118)	(0.116)	(0.119)	(0.116)	(0.119)	(0.116)	(0.139)	(0.135)
51-70 years	-0.510***	-0.532***	-0.522***	-0.551***	-0.539***	-0.545***	-0.558***	-0.566***
	(0.0998)	(0.0984)	(0.100)	(0.0986)	(0.101)	(0.0979)	(0.111)	(0.108)
**Gender**								
Identifies as female	0.113**	0.0772	0.0972*	0.0729	0.133**	0.0695	0.153**	0.0843
	(0.0544)	(0.0538)	(0.0551)	(0.0541)	(0.0550)	(0.0537)	(0.0743)	(0.0727)
Non-binary	-0.461	-0.348	-0.455	-0.339	-0.360	-0.311	-0.434	-0.508
	(0.487)	(0.480)	(0.488)	(0.479)	(0.495)	(0.482)	(1.121)	(1.092)
Identifies as Aboriginal and/or Torres Strait Islander	-0.310*	-0.295	-0.262	-0.292	-0.219	-0.268	-0.921***	-0.810**
	(0.186)	(0.183)	(0.187)	(0.183)	(0.188)	(0.183)	(0.348)	(0.339)
Has disability/ies	-1.025***	-1.008***	-1.018***	-1.018***	-1.013***	-1.029***	-0.918***	-0.993***
	(0.0970)	(0.0956)	(0.0977)	(0.0959)	(0.0972)	(0.0947)	(0.119)	(0.117)

Speaks language other than English at home	0.168*	0.151*	0.202**	0.159*	0.217**	0.155*	0.219	0.154
	(0.0921)	(0.0907)	(0.0925)	(0.0909)	(0.0932)	(0.0909)	(0.134)	(0.131)
**Highest level of education**								
Has not completed high school (Year 12)	0.205*	0.192*	0.220*	0.210*	0.160	0.169	0.157	0.172
	(0.118)	(0.116)	(0.119)	(0.117)	(0.118)	(0.115)	(0.152)	(0.148)
Certificate/Diploma	-0.0173	-0.0439	0.00131	-0.0275	0.00156	-0.0528	0.0207	-0.0233
	(0.0860)	(0.0847)	(0.0867)	(0.0851)	(0.0868)	(0.0846)	(0.119)	(0.116)
Undergraduate	0.0578	0.0498	0.102	0.0721	0.129	0.0620	0.0696	0.0313
	(0.0883)	(0.0870)	(0.0888)	(0.0872)	(0.0888)	(0.0866)	(0.125)	(0.122)
Postgraduate	0.149	0.145	0.199**	0.170*	0.251***	0.165*	0.145	0.0882
	(0.0945)	(0.0931)	(0.0950)	(0.0932)	(0.0949)	(0.0926)	(0.129)	(0.126)
**Employment status**								
Part-time	0.106	0.0813	0.109	0.0784	0.119	0.0749	0.187	0.144
	(0.0892)	(0.0879)	(0.0901)	(0.0884)	(0.0903)	(0.0880)	(0.126)	(0.123)
Casual	-0.129	-0.128	-0.126	-0.134	-0.139	-0.136	-0.473***	-0.460***
	(0.119)	(0.118)	(0.120)	(0.118)	(0.121)	(0.118)	(0.182)	(0.178)
Self-employed	0.256**	0.220**	0.230**	0.209*	0.255**	0.218*	0.193	0.187
	(0.114)	(0.112)	(0.115)	(0.112)	(0.115)	(0.112)	(0.150)	(0.146)
Engaged in home duties/volunteer work	0.375***	0.338***	0.355***	0.328**	0.359***	0.324**	0.259	0.241
	(0.129)	(0.127)	(0.130)	(0.128)	(0.131)	(0.127)	(0.174)	(0.169)
Retired	0.611***	0.574***	0.593***	0.565***	0.583***	0.556***	0.480***	0.457***
	(0.100)	(0.0990)	(0.101)	(0.0991)	(0.101)	(0.0986)	(0.121)	(0.118)
Not working/studying	-0.768***	-0.806***	-0.759***	-0.795***	-0.779***	-0.827***	-0.821***	-0.864***
	(0.165)	(0.162)	(0.167)	(0.164)	(0.167)	(0.163)	(0.221)	(0.215)
Student only	-0.513**	-0.501**	-0.495**	-0.485**	-0.353	-0.364*	-0.245	-0.191
	(0.217)	(0.214)	(0.218)	(0.214)	(0.217)	(0.211)	(0.427)	(0.416)
**Personal income quintile**								
Lowest income quintile	-0.524***	-0.544***	-0.492***	-0.523***	-0.524***	-0.558***	-0.593***	-0.621***
	(0.101)	(0.0993)	(0.102)	(0.0999)	(0.102)	(0.0994)	(0.133)	(0.129)
2nd-lowest income quintile	-0.171*	-0.179**	-0.167*	-0.183**	-0.173*	-0.198**	-0.166	-0.198*
	(0.0882)	(0.0869)	(0.0890)	(0.0873)	(0.0893)	(0.0870)	(0.119)	(0.116)
4th-highest income quintile	0.274***	0.263***	0.238***	0.244***	0.252***	0.243***	0.194*	0.174
	(0.0834)	(0.0822)	(0.0839)	(0.0824)	(0.0845)	(0.0823)	(0.117)	(0.114)
Highest income quintile	0.661***	0.661***	0.605***	0.644***	0.615***	0.644***	0.646***	0.632***
	(0.0953)	(0.0939)	(0.0957)	(0.0939)	(0.0965)	(0.0940)	(0.133)	(0.130)
Standardised IRSAD score	0.0442	0.0460	0.0548	0.0520	0.0543	0.0479	0.0335	0.0146
	(0.0343)	(0.0338)	(0.0345)	(0.0338)	(0.0346)	(0.0337)	(0.0454)	(0.0443)
**State/territory**								

Victoria	0.150	0.129	0.179*	0.159	0.155	0.136	0.162	0.171
	(0.101)	(0.0999)	(0.102)	(0.100)	(0.103)	(0.0998)	(0.125)	(0.122)
Queensland	0.132	0.116	0.140	0.123	0.102	0.0938	0.0769	0.0666
	(0.101)	(0.0999)	(0.102)	(0.0999)	(0.103)	(0.100)	(0.125)	(0.122)
South Australia	0.141	0.111	0.127	0.126	0.137	0.104	0.135	0.128
	(0.103)	(0.101)	(0.104)	(0.102)	(0.104)	(0.101)	(0.133)	(0.130)
Western Australia	0.0912	0.0811	0.0870	0.0973	0.0734	0.0693	0.0635	0.0689
	(0.102)	(0.101)	(0.103)	(0.101)	(0.103)	(0.101)	(0.130)	(0.127)
Tasmania	-0.0391	-0.0340	-0.0455	-0.0177	-0.0328	-0.0252	0.197	0.204
	(0.109)	(0.108)	(0.110)	(0.108)	(0.111)	(0.108)	(0.152)	(0.148)
Australian Capital Territory	0.0683	0.0717	0.0436	0.0803	0.0615	0.0663	0.336**	0.374**
	(0.109)	(0.108)	(0.110)	(0.108)	(0.111)	(0.108)	(0.150)	(0.147)
Northern Territory	-0.204	-0.212*	-0.175	-0.194	-0.157	-0.200	-0.154	-0.168
	(0.129)	(0.127)	(0.130)	(0.127)	(0.131)	(0.127)	(0.191)	(0.186)
**Current remoteness level**								
Regional	0.292***	0.271***	0.268***	0.256***	0.286***	0.257***	0.287***	0.254**
	(0.0776)	(0.0765)	(0.0783)	(0.0768)	(0.0784)	(0.0764)	(0.102)	(0.0995)
Remote	0.278*	0.221	0.216	0.175	0.230	0.189	0.331	0.314
	(0.157)	(0.155)	(0.159)	(0.156)	(0.159)	(0.155)	(0.223)	(0.218)
**Childhood remoteness level**								
Small/medium city	-0.0597	-0.0640	-0.0414	-0.0525	-0.0383	-0.0588	-0.00202	-0.0331
	(0.0711)	(0.0700)	(0.0716)	(0.0703)	(0.0719)	(0.0700)	(0.0967)	(0.0943)
Rural/regional	-0.0454	-0.0739	-0.00559	-0.0515	-0.0140	-0.0693	0.0163	-0.0269
	(0.0669)	(0.0660)	(0.0672)	(0.0661)	(0.0677)	(0.0660)	(0.0901)	(0.0879)
A mix	-0.449*	-0.451**	-0.415*	-0.433*	-0.440*	-0.499**	-0.413	-0.498*
	(0.232)	(0.228)	(0.232)	(0.228)	(0.233)	(0.227)	(0.301)	(0.293)
Constant	6.460***	5.534***	6.774***	5.608***	7.059***	5.513***	6.986***	5.481***
	(0.179)	(0.199)	(0.182)	(0.206)	(0.168)	(0.198)	(0.213)	(0.254)
Observations	3,445	3,445	3,421	3,421	3,481	3,481	2,020	2,020
R-squared	0.168	0.193	0.161	0.193	0.150	0.194	0.164	0.207
Pseudo R-squared			.	.	.	.	.	.

Note: *** significant at 1% level ** significant at 5% level * significant at 10% level. Regression coefficients shown, standard error in parentheses. Base case never engages in consumer-conscious behaviours, is aged over 70, identifies as male, does not identify as Aboriginal and/or Torres Strait Islander, does not have a disability, speaks only English at home, highest level of education is high school completion (Year 12 certificate), employed full time, is in the middle income quintile, currently lives in NSW, lives in a major city, and grew up in a large/capital city.

Full survey

SECTION A: DEMOGRAPHICS

Please tell us a little about yourself and your situation.

Age: Please specify your age:
1. __________ years

Gen: Please specify your gender:
1. Female
2. Male
3. Non-binary
4. Prefer not to answer
5. Other (specify): _____________

EmpStat: What is your current employment status? (If employed but currently on leave, this would still be classified as employed)
1. Employed full time (30 or more hours/week)
2. Employed part time (less than 30 hours/week)
3. Employed casually
4. Self-employed
5. Engaged in home duties or volunteer work
6. Retired
7. Unemployed
8: Other:

Enviro. Do you work in the environment sector?
1. Yes
2. No

Edu1: What is the highest level of education qualification you have completed thusfar?
1. Year 10 or below
2. Year 11
3. Year 12
4. Certificate I/II
5. Certificate III/IV
6. Diploma / Advanced Diploma
7. Bachelor’s degree
8. Graduate diploma / Graduate certificate
9. Postgraduate degree

Edu2: Are you currently studying towards a qualification?
Y/N
If Yes:
1. Year 10 or below

2. Year 11
3. Year 12
4. Certificate I/II
5. Certificate III/IV
6. Diploma / Advanced Diploma
7. Bachelor's degree
8. Graduate diploma / Graduate certificate
9. Postgraduate degree
10. Other: ____

Disab: Do you identify as having a disability?
1. Yes
2. No

ATSI: Do you identify as Aboriginal or Torres Strait Islander?
1. Yes, Aboriginal
2. Yes, Torres Strait Islander
3. Yes, Aboriginal and Torres Strait Islander
4. No
5. Prefer not to answer

LOTE: What is/are the main language(s) you speak at home?
1. Only English
2. Other(s) (SPECIFY):

COB: In which country were you born?
1. Australia
2. Other (SPECIFY):

*(IF COB=2) **ARR**: In what year did you arrive in Australia?
1. _________ (year)

CHE: What sort of environment did you grow up in (to age 16):
1. Rural/regional area (less than 50,000 people)
2. Small to medium city (50-250 thousand people)
3. Large or capital city (more than 250 thousand people)
4. A mix of these/other: (SPECIFY)

(ALL) **PI**: What is your approximate HOUSEHOLD income? This refers to the total income from all household occupants, and includes income from wages and salaries, government benefits, pensions, allowances and any other income you usually receive, before deductions for tax, superannuation contributions, health insurance, amounts salary sacrificed, or any other automatic deductions.
1. $1-$199 per week ($1-$10,399 per year)

2. $200-$299 per week ($10,400-$15,599 per year)
3. $300-$399 per week ($15,600-$20,799 per year)
4. $400-$599 per week ($20,800-$31,199 per year)
5. $600-$799 per week ($31,200-$41,599 per year)
6. $800-$999 per week ($41,600-$51,999 per year)
7. $1,000-$1,249 per week ($52,000-$64,999 per year)
8. $1,250-$1,499 per week ($65,000-$77,999 per year)
9. $1,500-$1,999 per week ($78,000-$103,999 per year)
10. $2,000 $2,499 per week ($104,000-$129,999 per year)
11. $2,500-$2,999 per week ($130,000-$155,999 per year)
12. $3,000-$3,499 per week ($156,000-$181,999 per year)
13. $3,500-$3,999 per week ($182,000-$207,999 per year)
14. $4,000-$4,999 per week ($208,000-$259,999 per year)
15. $5,000 or more per week ($260,000 or more per year)
16. Negative or nil income
17. Prefer not to answer

MarS: What is your current marital status?
1. Never married
2. Widowed
3. Divorced
4. Separated but not divorced
5. Married (in a 'de facto' or registered marriage)

ECHS. Are you a parent or guardian of a child or children (17 years or younger)?
1. Yes
2. No

Pcode: And what is the postcode of your main residence?
1. __________

SECTION B: Human-Nature Connection

In this section we will ask you questions about how connected you feel with nature and what 'human-nature connection' looks like for you.

ND1: What comes to mind when you think of 'nature'? Please describe in your own words. (optional) [OPEN TEXT]

ND2. What kind of interactions do you have with nature in your everyday life? Please describe in your own words. (optional) [OPEN TEXT]

ND3: Think of a meaningful experience you have had that shaped the way you think about 'nature'? Please describe it in 1-3 sentences in your own words. (optional) [OPEN TEXT]

CN1. INS scale: How (inter)connected are you with nature in general? Choose the picture which best describes your relationship to nature.

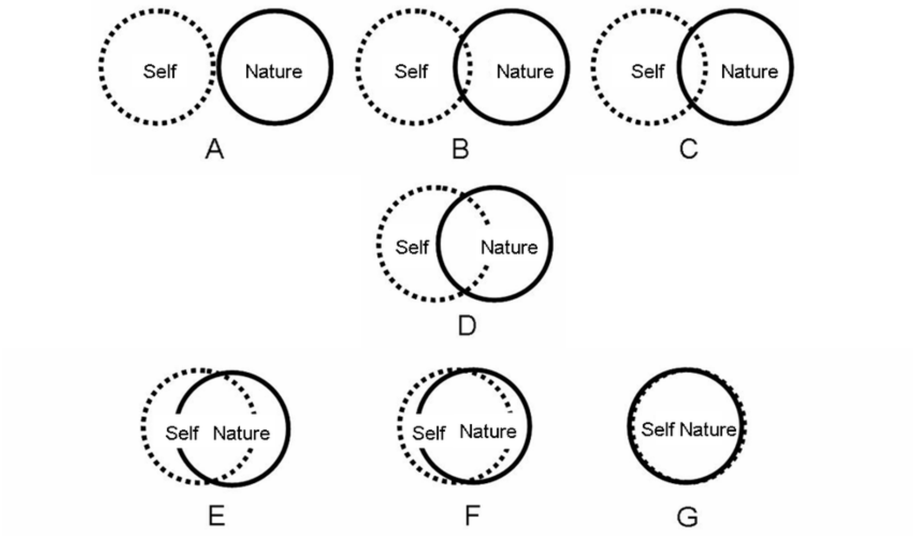

CN2. Please rate the extent to which you agree or disagree with the following statements:
[PROGRAMMER NOTE: RANDOMISE STATEMENTS]

**Strongly disagree (1)**	**(2)**	**(3)**	**Neither agree nor disagree (4)**	**(5)**	**(6)**	**Strongly agree (7)**

1. I think of myself as someone who is very concerned about taking care of nature
2. My relationship to nature is a big part of how I think about myself
3. I feel uneasy if I am away from nature for too long
4. I feel right at home when I am in nature
5. Feeling connected to nature helps me deal with everyday stress
6. I feel a strong emotional connection to nature
7. I enjoy spending time in nature
8. I like to get outdoors whenever I get the chance
9. Being in nature allows me to do the things I like doing most
10. Everything in nature is connected (e.g. animals, plants, humans, water, air, land, fire, etc.)
11. Human beings and nature are connected by the same ‘energy’ or ‘life-force’
12. Human wellbeing depends upon living in harmony with nature

SECTION C: Nature engagement and environmental behaviours

In this section we will ask you about what types of nature you interact with, what you do while you are there and how those interactions relate to other behaviours and values in your life.

EB1. Over the last year or two, about how often have you generally spent time in nature?
1. Never
2. Less than once a year
3. At least once a year
4. At least twice a year
5. At least once a month
6. At least once a fortnight
7. At least once a week
8. A couple times a week
9. Every day

EB2. Over the last year or two, how often have you spent time in/at the following places?
[PROGRAMMER NOTE: Pipe into EC1 and EC2]

NA (0)	Never (1)	Less than once a year (2)	At least once a year (3)	At least twice a year (4	At least once a month (5)	At least once a fortnig ht (6)	At least once a week (7)	A couple times a week (8)	Every day (9)

1. A forest (an area filled predominantly with tall trees)
2. A grassland/savanna/open woodland (an area with mostly grass or low vegetation with occasional trees, not managed for domestical animal grazing)
3. A desert/dry scrubland (a dry area with sand and rock and some hardy vegetation)
4. A lake, river or other inland waterways
5. A wetland, marsh, or estuary (Intermittent fresh water or is regularly flooded with fresh or salt water. They comprise swamps, marshes, billabongs, lakes, lagoons, bogs, fens and peatlands)
6. An agricultural area managed for crops or pasture
7. The beach or coastal areas
8. On or in the ocean water (as separate to the beach or coast)
9. A zoo or wildlife park
10. A grassy parkland, botanic garden or playing field (managed, grassy lawns, some trees, managed footpaths)
11. A community garden

12. 13. Your own garden or yard at home

EB3. Do you wish you could spend more time in nature? Y/N

If Yes, what are the major barriers to spending more time in nature? (select all that apply)

1. Too busy/not enough time

2. Lack of access to nature
3. Costs of accessing nature
4. Health issues prevent access
5. Friends and family aren't into nature
6. Safety concerns
7. Unsure of where to go
8. Other:

If no:
1. I don't like spending time in nature
2. I feel I spend enough time in nature
3. I think nature is scary or unsafe
4. Other: (specify)

PEB4: How often do you engage in the following environmental behaviours??

[randomize order]	Never (1)	Rarely (2)	Sometimes (3)	Often (4)	Very often (5)	NA
I bring up environmental issues in conversation with my peers						
I try to encourage others to change a behaviour that I think is harmful to the environment						
I bring up positive nature experiences in a conversation, such as interesting facts about wildlife or stories about things I've seen or done in nature						
I share articles, pictures, or videos on social media about nature or environmental issues						
I consider environmental impacts when making purchasing decisions (e.g. product/service waste, packaging, carbon footprint, sustainability, local source etc)						
I contact businesses or governments about impacts on environmental issues I'm concerned about						
I sign petitions about environmental issues I'm concerned about						
I donate money to organisations that protect or support the environment						
I attend protests or rallies related to environmental issues						
I vote for people, parties, or policies that support nature						

Section D: Wellbeing

In this section, we will ask about different aspects of your health and wellbeing including how satisfied you are with different aspects of your life. We want to understand both how different components of your wellbeing (e.g. physical, social, mental aspects) might benefit from nature engagement or connection and whether there are any aspects of your health that interfere with your engagement with nature.

Wel1: Thinking about your own life and personal circumstances, how satisfied are you with the following aspects of your life:

No satisfaction at all										Completely Satisfied
0	1	2	3	4	5	6	7	8	9	10
☐	☐	☐	☐	☐	☐	☐	☐	☐	☐	☐

1. your life as a whole?
2. your standard of living?
3. your health?
4. what you are achieving in life?
5. your personal relationships?
6. how safe you feel?
7. feeling part of your community?
8. your future security?
9. the quality of your local environment

Wel2. For these questions, please tick the box that best describes your situation as it has been <u>over the past week</u>
Q1 How much help do you need with jobs around your place of residence (eg preparing food, cleaning, gardening)?
☐ I can do all these tasks very quickly and efficiently without any help
☐ I can do these tasks relatively easily without help
☐ I can do these tasks only very slowly without help
☐ I cannot do most of these tasks unless I have help
☐ I can do none of these tasks by myself.

Q2 How easy or difficult is it for you to get around by yourself outside your place of residence (eg to go shopping, visiting)?
☐ getting around is enjoyable and easy
☐ I have no difficulty getting around outside my place of residence
☐ I have a little difficulty
☐ I have moderate difficulty
☐ I have a lot of difficulty
☐ I cannot get around unless somebody is there to help me.

Q3 How easy or difficult is it for you to move around (using any aids or equipment you need eg a wheelchair, frame or stick)?
☐ I am very mobile
☐ I have no difficulty with mobility
☐ I have some difficulty with mobility (for example, going uphill)
☐ I have difficulty with mobility. I can go short distances only.
☐ I have a lot of difficulty with mobility. I need someone to help me.
☐ I am bedridden.

Q4 How difficult is it for you to wash, toilet, dress yourself, eat or care for your appearance?
☐ these tasks are very easy for me
☐ I have no real difficulty in carrying out these tasks
☐ I find some of these tasks difficult, but I manage to do them on my own
☐ many of these tasks are difficult, and I need help to do them
☐ I cannot do these tasks by myself at all.

Q5 How happy are you with your close and intimate relationships?
☐ very happy
☐ generally happy
☐ neither happy nor unhappy
☐ generally unhappy
☐ very unhappy

Q6 Does your health affect your relationship with your family?
☐ my role in the family is unaffected by my health
☐ there are some parts of my family role I cannot carry out
☐ there are many parts of my family role I cannot carry out
☐ I cannot carry out any part of my family role.

Tick the box that best describes your situation as it has been over the past week
Q7 Does your health affect your role in your community (eg residential, sporting, church or cultural groups)?
☐ my role in the community is unaffected by my health
☐ there are some parts of my community role I cannot carry out
☐ there are many parts of my community role I cannot carry out
☐ I cannot carry out any part of my community role.

Q8 How often did you feel in despair over the last seven days?
☐ never
☐ occasionally
☐ sometimes
☐ often
☐ all the time.

Q9 How often did you feel worried in the last seven days?
☐ never
☐ occasionally
☐ sometimes
☐ often

☐ all the time.

Q10 How often do you feel sad?
☐ never
☐ rarely
☐ some of the time
☐ usually
☐ nearly all the time.

Q11 Do you normally feel calm and tranquil or agitated?
I am
☐ always calm and tranquil
☐ usually calm and tranquil
☐ sometimes calm and tranquil, sometimes agitated
☐ usually agitated
☐ always agitated.

Q12 How much energy do you have to do the things you want to do?
I am
☐ always full of energy
☐ usually full of energy
☐ occasionally energetic
☐ usually tired and lacking energy
☐ always tired and lacking energy.

Q13 How often do you feel in control of your life?
☐ always
☐ mostly
☐ sometimes
☐ only occasionally
☐ never.

Tick the box that best describes your situation as it has been over the past week
Q14 How much do you feel you can cope with life's problems?
☐ completely
☐ mostly
☐ partly
☐ very little
☐ not at all.

Q15 How often do you experience serious pain?
I experience it
☐ very rarely
☐ less than once a week
☐ three to four times a week
☐ most of the time.

Q16 How much pain or discomfort do you experience?
☐ none at all
☐ I have moderate pain
☐ I suffer from severe pain

□ I suffer unbearable pain.

Q17 How often does pain interfere with your usual activities?
□ never
□ rarely
□ sometimes
□ often
□ always

Q18 How well can you see (using your glasses or contact lenses if needed)?
□ I have excellent sight
□ I see normally
□ I have some difficulty focusing on things, or I do not see them sharply. *E.g. small print, a newspaper or seeing objects in the distance.*
□ I have a lot of difficulty seeing things. *My vision is blurred. I can see just enough to get by with.*
□ I only see general shapes. *I need a guide to move around*
□ I am completely blind.

Q19 How well can you hear (using your hearing aid if needed)?
□ I have excellent hearing
□ I hear normally
□ I have some difficulty hearing or I do not hear clearly. *I have trouble hearing softly-spoken people or when there is background noise.*
□ I have difficulty hearing things clearly. *Often I do not understand what is said. I usually do not take part in conversations because I cannot hear what is said.*
□ I hear very little indeed. *I cannot fully understand loud voices speaking directly to me.*
□ I am completely deaf.

Tick the box that best describes your situation as it has been over the past week
Q20 How well do you communicate with others (talking, signing, texting, being understood by others and understanding them)?
□ I have no trouble speaking to them or understanding what they are saying
□ I have some difficulty being understood by people who do not know me. I have no trouble understanding what others are saying to me.
□ I am understood only by people who know me well. I have great trouble understanding what others are saying to me.
□ I cannot adequately communicate with others.

SECTION E: Nature Connection and Wellbeing across environments

In this section, we ask how connected you feel to/at/with different environments and how much you feel those environments contribute to your wellbeing:

EC1.Please indicate how (inter)connected you feel to nature in/at/with the following places:

NA/I have never visited this place (0)	Not connected at all (1)	(2)	(3)	Somewhat connected (4)	(5)	(6)	Very strongly connected (7)

1. A forest (an area filled predominantly with tall trees)
2. A grassland/savanna/open woodland (an area with mostly grass or low vegetation with occasional trees, not managed for domestical animal grazing)
3. A desert/dry scrubland (a dry area with sand and rock and some hardy vegetation)
4. A lake, river or other inland waterways
5. A wetland, marsh, or estuary (Intermittent fresh water or is regularly flooded with fresh or salt water. They comprise swamps, marshes, billabongs, lakes, lagoons, bogs, fens and peatlands.)
6. An agricultural area managed for crops or pasture
7. The beach or coastal areas
8. On or in the ocean water (as separate to the beach or coast)
9. A zoo or wildlife park
10. A grassy parkland, botanic garden or playing field (managed, grassy lawns, some trees, managed footpaths)
11. A community garden
12. Your own garden or yard at home
13. Observing the night sky
14. 15. Other _____

EC1a. Think about the <u>environment</u> in which you feel most (inter)connected with nature. Can you describe what it is about that place that makes you feel (inter)connected with nature (e.g. are there features of that place or things you do there that help you connect etc)? (optional)
[open text]
EC1b. Think about the <u>times</u> in which you feel most (inter)connected with nature. Can you describe what it is about that time that makes you feel (inter)connected with nature (e.g. what are you doing, who are you with, what is happening around you)? (optional)
[open text]

EC2. Please indicate how much you feel each of these places directly benefits your personal health and wellbeing when you visit it: *[PROGRAMMER NOTE: DISPLAY ITEMS FROM EC2 =>1]*

NA/I have never visited this place (0)	No benefit to my wellbeing (1)	(2)	(3)	Some benefit to my wellbeing (4)	(5)	(6)	A great benefit to my wellbeing (7)

1. A forest (an area filled predominantly with tall trees)
2. A grassland/savanna/open woodland (an area with mostly grass or low vegetation with occasional trees, not managed for domestical animal grazing)

3. A desert/dry scrubland (a dry area with sand and rock and some hardy vegetation)
4. A lake, river or other inland waterways
5. A wetland, marsh, or estuary (Intermittent fresh water or is regularly flooded with fresh or salt water. They comprise swamps, marshes, billabongs, lakes, lagoons, bogs, fens and peatlands.)
6. An agricultural area managed for crops or pasture
7. The beach or coastal areas
8. On or in the ocean water (as separate to the beach or coast)
9. A zoo or wildlife park
10. A grassy parkland, botanic garden or playing field (managed, grassy lawns, some trees, managed footpaths)
11. A community garden
12. Your own garden or yard at home
13. Observing the night sky
14. Other

EC2a. Think about the environment which you feel most benefits your health and wellbeing. What kinds of experiences do you have that support your wellbeing when you visit this place? (optional)
[open text]

EC3. Please think about what you do when you have visited these different types of environments in the last year or two. Select all that apply. [Programmer note: Pipe this from EC1, exclude any marked ‘never’, or ‘NA’]

1. A forest

Rest and recover	Disconnect from daily life	Socialise	Accompany children to activities	Engage in physical activities	Engage in motorised leisure activities (e.g. dirt-biking, 4-wheeling, off-roading)	Experience a sense of peace, tranquillity or awe	Engage in cultural activities	Enjoy nature	Have a picnic or BBQ	Walk your pet	Pass through to reach your destination	Engage in activities to protect or care for nature (e.g. landcare, conservation)	Look up at the moon or stars in the sky	Go hunting	Go fishing	Learn about the plants, animals or environment (from books, apps, people etc)	Other

2. A grassland/savanna/open woodland

Rest and recover	Disconnect from daily life	Socialise	Accompany children to activities	Engage in physical activities	Engage in motorised leisure activities (e.g. dirt-biking, 4-wheeling, off-roading)	Experience a sense of peace, tranquillity or awe	Engage in cultural activities	Enjoy nature	Have a picnic or BBQ	Walk your pet	Pass through to reach your destination	Engage in activities to protect or care for nature (e.g. landcare, conservation)	Look up at the moon or stars in the sky	Go hunting	Go fishing	Learn about the plants, animals or environment (from books, apps, people etc)	Other

3. A desert/dry scrubland

Rest and recover	Disconnect from daily life	Socialise	Accompany children to activities	Engage in physical activities	Engage in motorised leisure activities (e.g. dirt-biking, 4-wheeling, off-roading)	Experience a sense of peace, tranquillity or awe	Engage in cultural activities	Enjoy nature	Have a picnic or BBQ	Walk your pet	Pass through to reach your destination	Engage in activities to protect or care for nature (e.g. landcare, conservation)	Look up at the moon or stars in the sky	Go hunting	Go fishing	Learn about the plants, animals or environment (from books, apps, people etc)	Other

4. A lake, river or other inland waterway

Rest and recove r	Disconne ct from daily life	Socialis e	Accompa ny children to activities	Engage in physica l activitie s	Engage in motorise d leisure activities (e.g. boating, dirt-biking, off-roading)	Experienc e a sense of peace, tranquillit y or awe	Engage in cultural activitie s	Enjoy natur e	Have a picni c or BBQ	Wal k your pet	Pass through/b y to reach your destinatio n	Engage in activities to protect or care for nature (e.g. landcare, conservatio n)	Look up at the moo n or stars in the sky	Go huntin g	Go fishin g	Learn about the plants, animals or environme nt (from books, apps, people etc)	Othe r

5. A wetland, marsh, or estuary

Rest and recover	Disconnect from daily life	Socialise	Accompany children to activities	Engage in physical activities	Engage in motorised leisure activities (e.g. boating, dirt-biking, off-roading)	Experience a sense of peace, tranquillity or awe	Engage in cultural activities	Enjoy nature	Have a picnic or BBQ	Walk your pet	Pass through to reach your destination	Engage in activities to protect or care for nature (e.g. landcare, conservation)	Look up at the moon or stars in the sky	Go hunting	Go fishing	Learn about the plants, animals or environment (from books, apps, people etc)	Other

6. An agricultural area

Rest and recover	Disconnect from daily life	Socialise	Accompany children to activities	Engage in physical activities	Engage in motorised leisure activities (e.g. e.g. dirt-biking, 4-wheeling, off-roading))	Experience a sense of peace, tranquillity or awe	Engage in cultural activities	Enjoy nature	Have a picnic or BBQ	Walk your pet	Gardening activities	Pass through to reach your destination	Engage in activities to protect or care for nature (e.g. landcare, conservation)	Look up at the moon or stars in the sky	Go hunting	Go fishing	Learn about the plants, animals or environment (from books, apps, people etc)	Other

7. The beach or coastal areas

Rest and recover	Disconnect from daily life	Socialise	Accompany children to activities	Engage in physical activities	Engage in motorised leisure activities (e.g. e.g. dirt-biking, 4-wheeling, off-roading)	Experience a sense of peace, tranquillity or awe	Engage in cultural activities	Enjoy nature	Have a picnic or BBQ	Walk your pet	Pass through to reach your destination	Engage in activities to protect or care for nature (e.g. coast care, conservation, marine debris cleanup)	Look up at the moon or stars in the sky	Go hunting	Go fishing	Learn about the plants, animals or environment (from books, apps, people etc)	Other

8. On or in the ocean water (i.e. as separate to the beach or coast)

Rest and recover	Disconnect from daily life	Socialise	Accompany children to activities	Engage in physical activities	Engage in motorised leisure activities (e.g. boating, Jet skiing)	Experience a sense of peace, tranquillity or awe	Engage in cultural activities	Enjoy nature	Have a picnic or BBQ	Walk your pet	Engage in activities to protect or care for nature (e.g. marine debris cleanup, starfish removal)	Look up at the moon or stars in the sky	Go fishing	Learn about the plants, animals or environment (from books, apps, people etc)	Other

9. A zoo or wildlife park

Rest and recover	Disconnect from daily life	Socialise	Accompany children to activities	Engage in physical activities	Experience a sense of peace, tranquillity or awe	Engage in cultural activities	Enjoy nature	Have a picnic or BBQ	Walk your pet	Engage in activities to protect or care for nature (e.g. landcare, conservation)	Look up at the moon or stars in the sky	Go hunting	Go fishing	Learn about the plants, animals or environment (from books, apps, people etc)	Other

10. A botanic garden or urban park with grassy lawns and trees

Rest and recover	Disconnect from daily life	Socialise	Accompany children to activities	Engage in physical activities	Experience a sense of peace, tranquillity or awe	Engage in cultural activities	Enjoy nature	Have a picnic or BBQ	Walk your pet	Pass through to reach your destination	Engage in activities to protect or care for nature (e.g. landcare, conservation)	Look up at the moon or stars in the sky	Go hunting	Go fishing	Learn about the plants, animals or environment (from books, apps, people etc)	Other

11. A community garden

Rest and recover	Disconnect from daily life	Socialise	Accompany children to activities	Engage in physical activities	Experience a sense of peace, tranquillity or awe	Engage in cultural activities	Enjoy nature	Have a picnic or BBQ	Walk your pet	Gardening or lawncare activities	Pass through to reach your destination	Engage in activities to protect or care for nature (e.g. landcare, conservation)	Look up at the moon or stars in the sky	Learn about the plants, animals or environment (from books, apps, people etc)	Other

12. Your own garden or yard at home

Rest and recover	Disconnect from daily life	Socialise	Accompany children to activities	Engage in physical activities	Engage in motorised leisure activities (e.g. dirt-biking, 4-wheeling, off-roading)	Experience a sense of peace, tranquillity or awe	Engage in cultural activities	Enjoy nature	Have a picnic or BBQ	Walk your pet	Gardening or lawncare activities	Pass through to reach your destination	Engage in activities to protect or care for nature (e.g. conservation, mange for biodiversity, create habitat wildlife)	Look up at the moon or stars in the sky	Go hunting	Go fishing	Learn about the plants, animals or environment (from books, apps, people etc)	Other
